# DNA nicks in both leading and lagging strand templates can trigger break-induced replication

**DOI:** 10.1016/j.molcel.2024.10.026

**Published:** 2025-01-02

**Authors:** Yuanlin Xu, Carl A. Morrow, Yassine Laksir, Orla M. Holt, Kezia Taylor, Costas Tsiappourdhi, Patrick Collins, Su Jia, Christos Andreadis, Matthew C. Whitby

**Affiliations:** 1 Department of Biochemistry, University of Oxford, South Parks Road, Oxford OX1 3QU, UK

**Keywords:** single-strand break, DNA replication, homologous recombination, replication fork, replication fork collapse, replication restart, break-induced replication, CRISPR-Cas9n, Flp-nick, double-strand break

## Abstract

Encounters between replication forks and unrepaired DNA single-strand breaks (SSBs) can generate both single-ended and double-ended double-strand breaks (seDSBs and deDSBs). seDSBs can be repaired by break-induced replication (BIR), which is a highly mutagenic pathway that is thought to be responsible for many of the mutations and genome rearrangements that drive cancer development. However, the frequency of BIR’s deployment and its ability to be triggered by both leading and lagging template strand SSBs were unclear. Using site- and strand-specific SSBs generated by nicking enzymes, including CRISPR-Cas9 nickase (Cas9n), we demonstrate that leading and lagging template strand SSBs in fission yeast are typically converted into deDSBs that are repaired by homologous recombination. However, both types of SSBs can also trigger BIR, and the frequency of these events increases when fork convergence is delayed and the non-homologous end joining protein Ku70 is deleted.

## Highlights

• Replication of nicked DNA mostly causes two-ended DSBs that are faithfully repaired
• Leading and lagging strand template nicks induce BIR-associated template switching
• Nick-induced BIR is promoted by Rad51, Pfh1^Pif1^, and Cdc27^Pol32/POLD3^
• Nick-induced BIR is suppressed by Ku70 and the converging replication fork

## Graphical abstract

**Figure:**
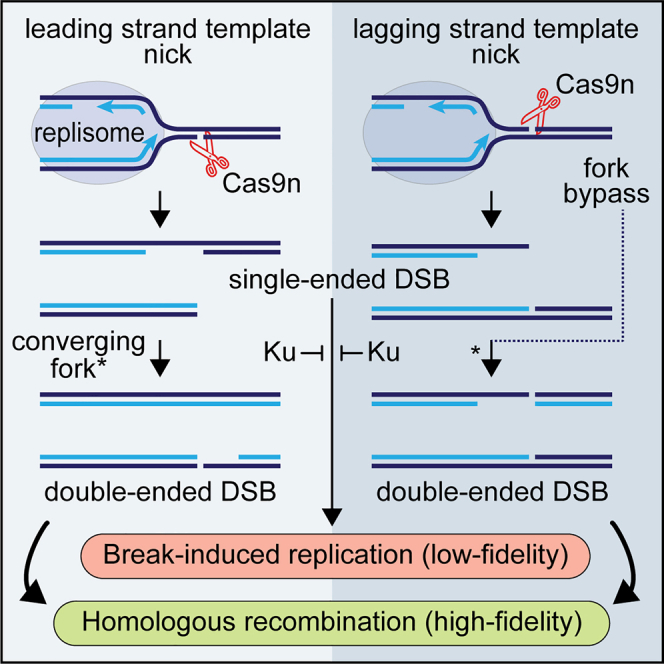

## Teaser

Xu et al. demonstrate that single-stranded breaks in either the leading or lagging template DNA strand can initiate break-induced replication in fission yeast. Various nicking enzymes, including Cas9n, provoke characteristic break-induced replication repair products, which are suppressed by the Ku complex and the oncoming replication fork.

## Introduction

DNA single-strand breaks (SSBs) are among the most pervasive DNA lesions found in cells, occurring at a frequency of 10^4^–10^5^ per cell per day.^1^ These breaks can result from ionizing radiation and reactive oxygen species and are formed as intermediates in several DNA metabolic processes, including base excision repair and DNA torsional stress relief by topoisomerase I (Top1).^1^^,^^2^ Because SSBs are so numerous, they can sometimes be encountered by DNA replication forks before they are repaired.^1^^,^^2^ These encounters can cause genome instability and cytotoxicity and also form the basis of cancer treatments that use poly(ADP-ribose) polymerase (PARP) inhibitors and derivatives of the Top1 poison camptothecin (CPT) to increase SSB accumulation in cells.^3^^,^^4^^,^^5^

Insights into the behavior of replication forks encountering SSBs have partly come from studies of CPT-induced breaks. CPT inhibits the re-ligation step in Top1’s catalytic cycle, leading to the formation of a cleavage complex (Top1cc) that consists of an SSB with Top1 covalently attached at its 3′ end.^3^ Initial studies showed that CPT could cause replication-associated DNA double-strand breaks (DSBs).^6^^,^^7^^,^^8^ It was later demonstrated that DSBs arise through replication run-off, where the replicative helicase CDC45-MCM2-7-GINS (CMG) translocates off the DNA at Top1ccs in the leading strand template.^9^ Further studies using DNA-nicking enzymes to create site-specific SSBs confirmed that SSBs can be converted into either single-ended DSBs (seDSBs) or double-ended DSBs (deDSBs) during DNA replication.^10^^,^^11^^,^^12^^,^^13^^,^^14^ Recent biochemical experiments in *Xenopus* egg extract, along with END-seq analysis in mammalian cells, verified that seDSBs are generated by replication run-off at leading strand template SSBs (lead-SSBs), which can then be converted into deDSBs through fork convergence.^15^^,^^16^ However, contrasting fates were observed for replication forks encountering SSBs in the lagging strand template (lag-SSBs).^15^^,^^16^ When lag-SSBs were introduced using the CRISPR-Cas9 nickase (Cas9n) variant H840A, seDSBs were observed.^15^^,^^16^ By contrast, lag-SSBs created by the Cas9n D10A variant predominantly resulted in deDSBs.^15^

Similar to other hexameric helicases, CMG unwinds DNA via steric exclusion of the strand on which it is not translocating, which in this context is the lagging strand template.^17^^,^^18^ However, at fully base-paired single-strand DNA (ssDNA) to double-strand DNA (dsDNA) junctions, strand exclusion fails and CMG translocates onto the dsDNA.^18^^,^^19^ *In vivo*, this serves as a cue for the active unloading of CMG from the DNA via a ubiquitin-mediated process, a mechanism that appears to be responsible for the generation of seDSBs at lag-SSBs made by Cas9n^H840A^.^16^^,^^20^^,^^21^^,^^22^ However, at lag-SSBs induced by Cas9n^D10A^, CMG seems to translocate past the DNA nick while continuing to exclude the lagging strand template.^15^ This replication bypass of a lag-SSB leads to the formation of a deDSB.

Only a few studies have investigated the repair of DSBs arising from replication at site-specific SSBs, with most analyzing plasmid substrates where the converging replication fork meets the already broken fork to form a deDSB.^13^^,^^23^^,^^24^ A key conclusion from these studies is that deDSBs that derive from replication bypass or run-off and fork convergence are predominantly repaired by homologous recombination (HR) through sister chromatid recombination (SCR). Key steps in this process include (1) nucleolytic resection of the break to generate 3′ ended ssDNA tails; (2) nucleation of the Rad51 recombinase onto the ssDNA, mediated by Rad52 in yeast or BRCA2 in humans; (3) Rad51 spreading along the DNA to form a nucleoprotein filament; (4) a Rad51-mediated search for a homologous dsDNA; and (5) invasion of the filament into the homologous dsDNA to establish a displacement- (D-) loop, priming new DNA synthesis. If the DSB is double-ended, these initial steps can lead to repair via synthesis-dependent strand annealing (SDSA) or through the formation and subsequent resolution/dissolution of a double Holliday junction.^25^^,^^26^^,^^27^ However, if the break is single-ended, a different HR mechanism, known as break-induced replication (BIR), comes into play.

BIR proceeds via continued elongation of the invading strand by a DNA polymerase coupled with migration of the D-loop.^28^^,^^29^ This non-canonical form of DNA replication can copy an entire chromosome arm but is highly mutagenic and prone to template switching, generating various genome rearrangements that are thought to drive the development of genetic diseases, including cancer.^28^^,^^29^^,^^30^^,^^31^ Given its potential to disrupt genomes and cause disease, it remains uncertain whether broken replication forks are typically repaired through BIR. Indeed, BIR may be reserved for specific circumstances where alternative repair mechanisms and means for completing DNA replication are unavailable, such as when cells experience oncogene-induced replication stress or enter mitosis with unreplicated DNA.^32^^,^^33^^,^^34^^,^^35^^,^^36^

So far, only one study has directly investigated whether replication run-off at SSBs induces BIR. This research, conducted in budding yeast, confirmed that SSBs can indeed trigger BIR.^14^ However, it remains unclear if BIR is a common and conserved response to replication run-off, how the nature of the SSB affects pathway choice, and whether both lead- and lag-SSBs can induce BIR. In this study, we investigate the ability of lead- and lag-SSBs to induce BIR in the fission yeast *Schizosaccharomyces pombe*, which is evolutionarily distant from budding yeast, similar to the relationship between yeast and humans.^37^ Using site- and strand-specific SSBs generated by Cas9n, we demonstrate that most SSBs are converted into deDSBs repaired through Rad51-mediated SCR. However, we also find that BIR can be induced from both lead- and lag-SSBs, with increased propensity for BIR when fork convergence is delayed and the non-homologous end joining (NHEJ) protein Ku70 is deleted.

## Results

### Cas9n-induced SSBs are converted into deDSBs that induce direct repeat recombination

**Figure 1:**
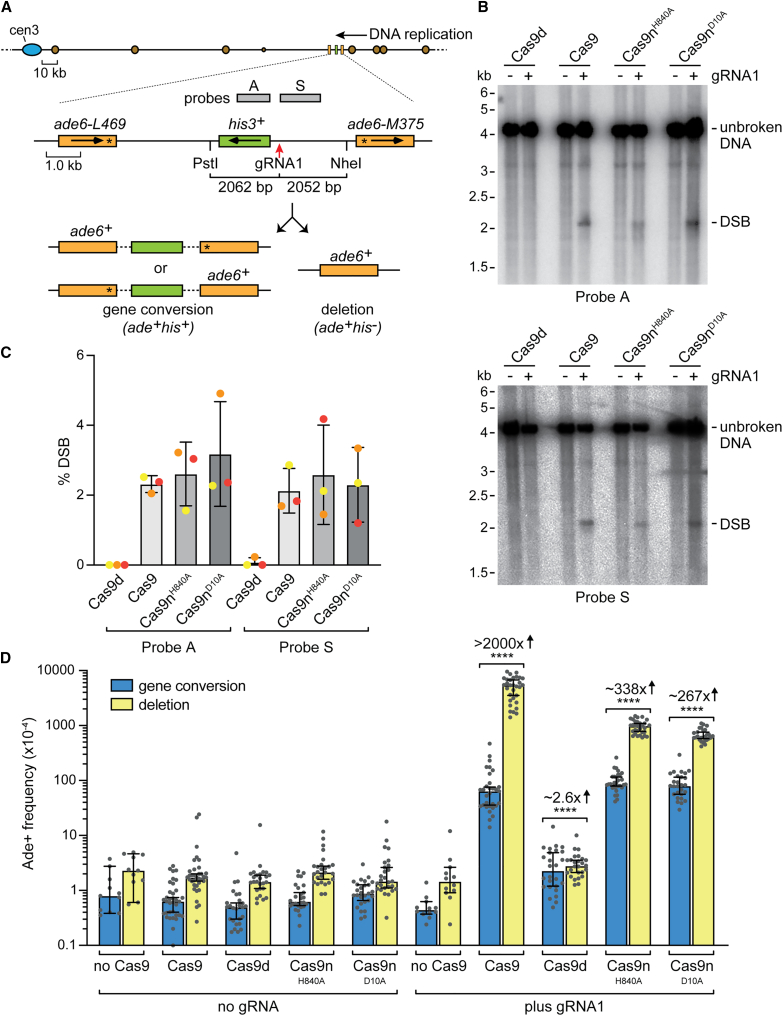
Cas9n-induced SSBs are converted into deDSBs that induce direct repeat recombination (A) Diagram showing the location of the recombination reporter and gRNA1 binding site (red arrow) on chromosome 3. It includes the centromere (blue oval), replication origins (brown circles), probes (gray rectangles), relevant restriction sites, and genes (orange and green rectangles with transcription direction indicated). The replication origins are sourced from the *S. pombe* DNA replication origin database: http://www.pombe.oridb.org. Asterisks mark the positions of loss-of-function mutations in the two *ade6* alleles. The bottom panel displays the two types of Ade+ recombinants. (B) Detection of DSBs in genomic DNA by Southern blot analysis. DNA was extracted from cells expressing the indicated version of Cas9 with and without gRNA1. The DNA was cut with PstI and NheI, and the relevant restriction fragment was detected using probes A and S (see A). (C) Quantification of the DSB signals detected by Southern blot analysis. Data are presented as mean values ± SD. Individual data points are shown as colored dots, with the color denoting which experiment the data are from. (D) Frequency of Ade+ recombinants in strains containing the *ade6*^*−*^ direct repeat substrate shown in (A). The presence of gRNA1 and version of Cas9 is indicated. Data are presented as median values ± 95% confidence interval with individual data points shown as gray dots. Fold changes and *p* values refer to the comparison of total Ade+ frequencies between the same strains with and without gRNA1. The *p* values were calculated by the two-tailed Mann-Whitney test. ^∗∗∗∗^*p* < 0.0001. See also Table S1.

To investigate the ability of lead- and lag-SSBs to generate DSBs and stimulate recombination, we designed guide RNA 1 (gRNA1) to direct Cas9n to make an SSB at a direct repeat recombination reporter integrated at the *ade6* locus on chromosome 3 of *S. pombe* (Figure 1A). At this site, DNA replication proceeds almost entirely in the telomere to centromere direction.^38^ We employed two Cas9n variants, D10A and H840A, to introduce strand-specific nicks. In our setup, gRNA1 binds to the lagging strand template, directing Cas9n^H840A^ to generate a lead-SSB and Cas9n^D10A^ to create a lag-SSB. To determine if these SSBs produce DSBs, we examined genomic DNA from asynchronously growing cell cultures using native gel electrophoresis, followed by Southern blot analysis to identify a 4.1 kb PstI-NheI restriction fragment encompassing the gRNA1 binding site (Figures 1A and 1B). As expected, cells expressing gRNA1 with fully active Cas9 showed DNA bands of ∼2 kb using probes that hybridize to either side of the gRNA binding site (Figure 1B). The percentage of both DSB signals was similar, consistent with them corresponding to the two sides of a deDSB (Figure 1C). No DSB signals were detected in genomic DNA from cells expressing the catalytically dead Cas9 (Cas9d) containing both D10A and H840A mutations (Figures 1B and 1C). Notably, genomic DNA from cells expressing either Cas9n^H840A^ or Cas9n^D10A^ with gRNA1 displayed similar levels of deDSB signals as those observed with Cas9 (Figures 1B and 1C). Consistent with other studies, the appearance of Cas9n-induced DSBs coincided with S phase, indicating that DNA replication converts SSBs into DSBs (Figure S1).^15^^,^^39^

To measure the recombination induced by Cas9 and Cas9n, we monitored the frequency of Ade+ recombinants arising from gene conversion and deletion recombination events between the two alleles of *ade6*^*−*^ in the reporter (Figures 1A and 1D). All three active forms of Cas9 induced high levels of direct repeat recombination, predominantly yielding deletions (Figure 1D). By contrast, Cas9d induced much lower levels of gene conversions and deletions (Figure 1D). Intriguingly, while the quantities of deDSBs and frequency of gene conversions induced by each active form of Cas9 were comparable, Cas9 induced ∼6–9 times more deletions than either version of Cas9n (Figure 1D; Table S1). We show later that Cas9-induced DSBs undergo frequent mutagenic repair. This would prevent site recleavage, leading to a lower level of DSB detection and an underestimation of the actual cleavage rate. Therefore, the higher frequency of Cas9-induced deletions may result from a greater DSB formation rate. Alternatively, deDSBs induced indirectly by Cas9n might be less prone to causing deletions than those induced directly by Cas9.

### DSBs arising from Cas9n-induced SSBs are primarily repaired through Rad51-dependent SCR

**Figure 2:**
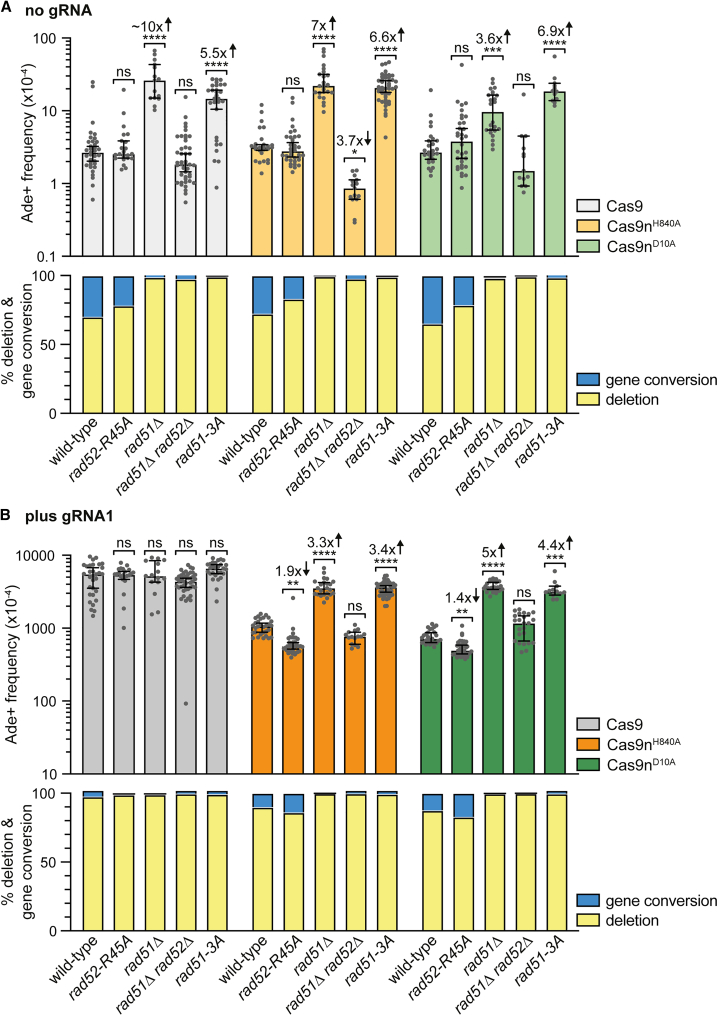
DSBs arising from Cas9n-induced SSBs are primarily repaired through Rad51-dependent SCR Effect of Cas9/Cas9n expression, without (A) and with (B) gRNA1, on the frequency of Ade+ recombinants in strains containing the *ade6*^*−*^ direct repeat substrate shown in Figure 1A. The top panel shows the frequency of Ade+ recombinants for each strain with data presented as median values ± 95% confidence interval with individual data points shown as gray dots. The bottom panel shows the percentage of deletions and gene conversions among Ade+ recombinants. Fold changes and *p* values are relative to the recombination value for the corresponding wild-type strain. The *p* values were calculated by the Kruskal-Wallis test with Dunn’s multiple comparisons post-test. ^∗∗∗∗^*p* < 0.0001; ^∗∗∗^*p* < 0.001; ^∗∗^*p* < 0.01; n.s., not significant. See also Table S1.

To determine if Cas9n-induced direct repeat recombination relies on the core HR proteins Rad51 and Rad52, we measured the frequency of Ade+ recombinants in strains lacking these proteins (Figure 2). Both spontaneous (no gRNA) and Cas9n-induced recombination (plus gRNA1) showed over a 3-fold increase in Ade+ deletions in a *rad51*Δ mutant compared with wild type, while the frequency of gene conversions decreased (Figure 2; Table S1). This aligns with Rad51’s role in promoting gene conversion through strand invasion and suppressing the single-strand annealing (SSA) pathway that leads to deletions (Figure S2A).^40^^,^^41^^,^^42^^,^^43^^,^^44^ SSA occurs when DNA resection exposes ssDNA at flanking repeats, allowing them to anneal and repair the DSB at the expense of deleting the interstitial DNA.^26^ As well as mediating the loading of Rad51 onto ssDNA, Rad52 also promotes SSA by annealing complementary ssDNAs.^45^^,^^46^ The increased deletion frequency in *rad51*Δ mutants depends on Rad52, suggesting that these recombinants are formed by SSA (Figure 2; Table S1). However, Cas9n-induced deletions were not completely eliminated in a *rad51*Δ *rad52*Δ double mutant, indicating that some DSBs can still be repaired via a deletion pathway independent of Rad51 and Rad52. Previous studies suggest that SSA is not entirely reliant on Rad52,^42^^,^^44^^,^^47^^,^^48^^,^^49^ so some observed Cas9n-induced deletions may arise from Rad52-independent SSA.

To determine if Rad52’s ssDNA annealing activity is necessary for spontaneous and Cas9n-induced deletions when Rad51 is active, we measured the frequency of Ade+ recombinants in a *rad52*-R45A mutant, which is defective in DNA annealing but retains Rad51-dependent recombination.^50^^,^^51^^,^^52^^,^^53^ The overall frequency of spontaneous recombinants and Cas9n-induced gene conversions remained unchanged in the *rad52*-R45A mutant. However, Cas9n-induced deletions decreased by nearly 2-fold, indicating that some DSBs may be repaired by Rad52-mediated SSA, even in the presence of Rad51 (Figures 2A and 2B; Table S1). To assess if Rad51 binding to DNA is sufficient to inhibit SSA, we measured the recombination frequency in a *rad51*-R152A-R324A-K334A (*rad51*-3A) mutant, which can form nucleoprotein filaments but is defective in strand invasion (Figure 2).^54^^,^^55^ This mutant showed a similar increase in spontaneous and Cas9n-induced deletions as the *rad51*Δ mutant, suggesting that Rad51 does not suppress Rad52-mediated SSA by directly blocking DNA annealing.

Taken together, these data indicate that deDSBs resulting from replication forks encountering lead- and lag-SSBs are primarily repaired by Rad51-dependent SCR (Figure S2A). This mechanism diverts repair away from the SSA pathway, ensuring most DSBs are repaired faithfully. This is possible because replication bypass or run-off at an SSB generates one broken and one mostly intact sister chromatid. Crucially, the intact sister chromatid serves as the necessary template for accurate repair. Unlike Cas9n, Cas9 can generate DSBs in both sister chromatids, precluding faithful SCR. Consequently, DSB repair under these conditions relies primarily on unequal sister chromatid exchange, SSA, NHEJ, or alternative end joining (aEJ) (Figure S2B).^56^^,^^57^ Notably, the high levels of Ade+ deletion recombinants induced by Cas9 were unaffected by the deletion or mutation of *rad51* and/or *rad52*, further supporting that DSB repair can occur via a Rad52-independent SSA pathway (Figure 2B). However, since our assay only detects recombinants in viable cells, this does not imply that Rad52-independent SSA is as efficient at repairing DSBs as the Rad51 and Rad52-dependent pathways.

### Cas9n-induced SSBs in the *kan*^R^ gene are less cytotoxic and mutagenic than DSBs induced by Cas9

**Figure 3:**
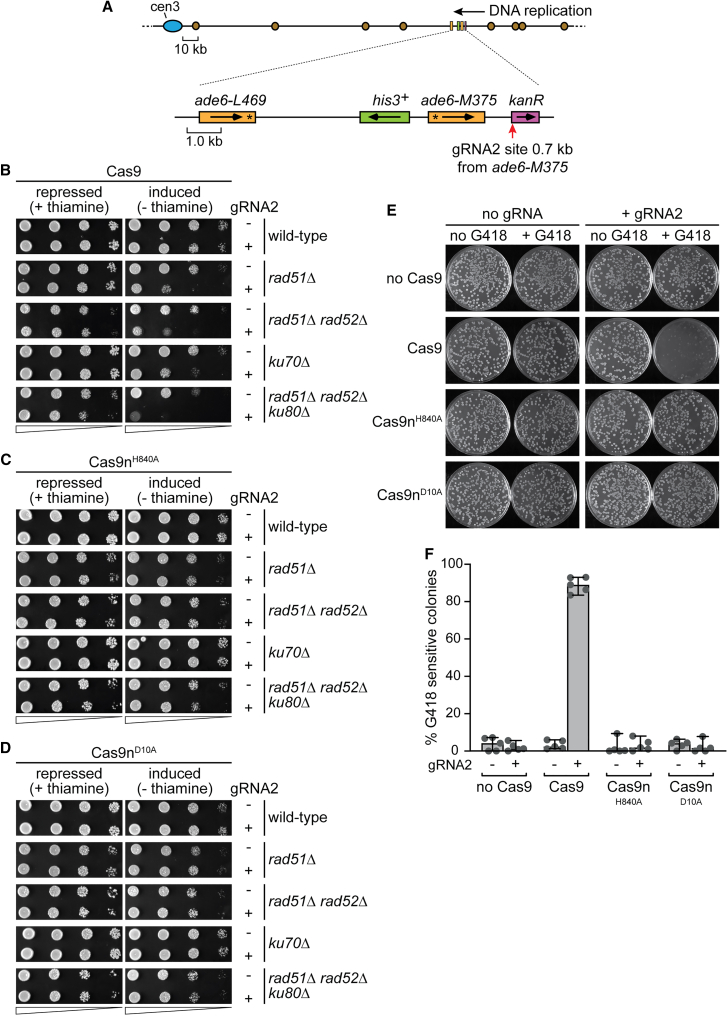
The cytotoxicity and mutagenicity of Cas9/Cas9n-induced DNA breaks (A) Diagram depicting the location of *kan*^*R*^ gene (purple rectangle) and the gRNA2 binding site (red arrow) on chromosome 3. An *ade6*^*−*^ direct repeat recombination reporter, similar to Figure 1A, is positioned 0.7 kb downstream of *kan*^*R*^. It also shows the centromere (blue oval), replication origins (brown circles), *ade6* genes (orange rectangles), and the *his3* gene (green rectangle), with arrows indicating transcription direction. Asterisks mark the positions of loss-of-function mutations in the two *ade6* alleles. (B) Spot assay showing the sensitivity of wild-type and indicated mutant strains to Cas9 cleavage of the *kan*^*R*^ gene shown in (A). In each case, sensitivity is compared with and without gRNA2, and with Cas9 expression repressed (+thiamine) or induced (−thiamine). (C) The same as (B) except sensitivity to Cas9n^H840A^-induced nicking of *kan*^*R*^ is shown. (D) The same as (B) except sensitivity to Cas9n^D10A^-induced nicking of *kan*^*R*^ is shown. (E) Comparison of *kan*^*R*^ gene mutation by Cas9 and Cas9n. Colonies, expressing the indicated version of Cas9, were grown with and without gRNA2. The colonies were then replica plated onto media with and without G418. The images show example replica plates after incubation at 30°C for 24 h. (F) Percentage of G418-sensitive colonies obtained from the plating assay shown in (E). Data from 5 independent experiments are presented as mean values ± SD. Individual data points are shown as gray dots.

To further investigate the repair of DSBs that arise from replication forks encountering Cas9n-induced SSBs, we designed a new gRNA (gRNA2) targeting the 5′ end of a *kan*^*R*^ gene, ∼0.7 kb from a recombination reporter located ∼12.4 kb centromere proximal to the site used in Figure 1A (Figure 3A). DNA replication of this locus initiates mainly from origins on the telomere proximal side of *kan*^*R*^. Therefore, since gRNA2 binds to the bottom strand, we can generate lead-SSBs with Cas9n^H840A^ and lag-SSBs with Cas9n^D10A^. This setup also allows us to assess the repair of Cas9/Cas9n-induced DSBs when SSA between *ade6* repeated DNA sequences is not possible.

Upon Cas9 induction in the presence of gRNA2, both *rad51*Δ single and *rad51*Δ *rad52*Δ double mutants exhibited a marked reduction in viability compared with wild type (Figure 3B). A reduction in viability was also observed when the gene encoding the NHEJ protein Ku70 was deleted (Figure 3B). However, the greatest reduction in viability was seen in a *rad51*Δ *rad52*Δ *ku80*Δ triple mutant that is defective in both HR and NHEJ (Figure 3B). In contrast to Cas9, Cas9n^H840A^ and Cas9n^D10A^ caused little or no reduction in viability in any of these mutants (Figures 3C and 3D). These data indicate that Cas9-induced DSBs are cytotoxic in the absence of effective repair by HR and NHEJ, whereas lead-SSBs and lag-SSBs in *kan*^R^ generated by Cas9n can be tolerated even when HR and NHEJ are non-functional.

The faithful repair of a DSB made by Cas9 will maintain its gRNA binding site and lead to repeated rounds of DNA breakage and repair until the site is mutated and can no longer be cleaved. The *kan*^*R*^ gene confers resistance to the antibiotic geneticin (G418), so we measured the number of G418-sensitive colonies to determine the frequency of Cas9/Cas9n-induced mutations (Figures 3E and 3F). Expression of Cas9, along with gRNA2, led to ∼90% of colonies becoming sensitive to G418 due to mutations at the Cas9 cleavage site. By contrast, neither version of Cas9n resulted in an increase in the frequency of G418-sensitive colonies (Figures 3E and 3F). We next compared the frequency of Cas9/Cas9n-induced G418-sensitive colonies in *rad51*Δ, *rad52*Δ, and *ku70*Δ/*ku80*Δ single, double, and triple mutants (Table S2). The frequency of Cas9-induced G418-sensitive colonies was noticeably higher in both *rad51*Δ and *ku70*Δ mutants than in wild type. However, none of the mutants tested exhibited detectable levels of Cas9n-induced G418-sensitive colonies. Based on these findings, and consistent with previous studies, we conclude that Cas9-induced DSBs can be repaired faithfully by both HR and NHEJ but are also subject to mutagenic repair.^56^^,^^57^ The absence of detectable levels of Cas9n-induced mutations in strains defective for HR and NHEJ, alongside the lack of Cas9n-induced cytotoxicity, leads us to suspect that lead- and lag-SSBs generated by Cas9n in *kan*^*R*^ are seldom converted into DSBs by DNA replication.

### Cas9n-induced SSBs can trigger BIR

**Figure 4:**
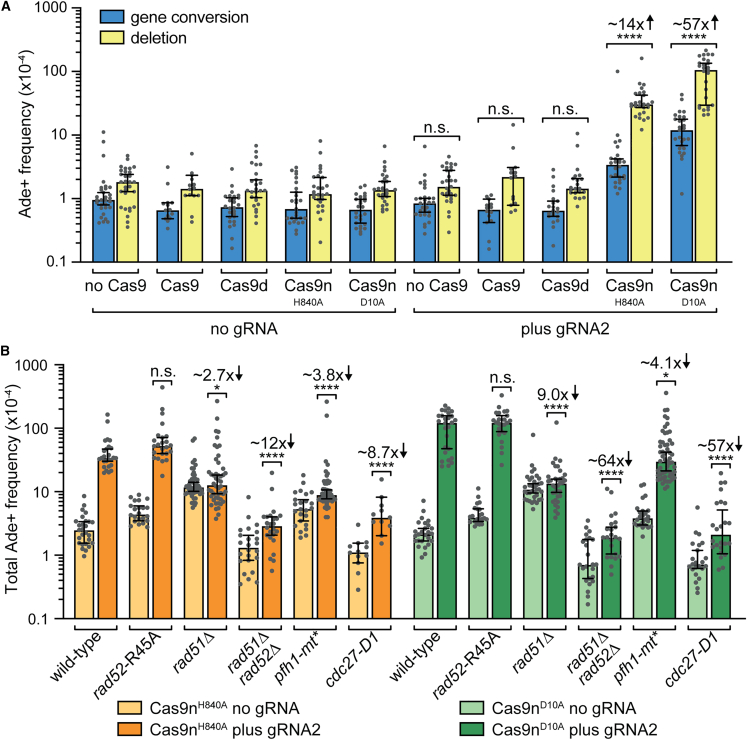
Cas9n-induced lead- and lag-SSBs trigger BIR (A) Effect of Cas9/Cas9n/Cas9d expression, with and without gRNA2, on the frequency of Ade+ recombinants in strains containing the *ade6*^*−*^ direct repeat recombination reporter and *kan*^*R*^ gene shown in Figure 3A. (B) Frequency of Cas9n-induced Ade+ recombinants in the indicated mutant strains containing the recombination reporter and *kan*^*R*^ gene shown in Figure 3A. In both (A) and (B), data are presented as median values ± 95% confidence interval with individual data points shown as gray dots. In (A), the indicated fold changes and *p* values relate to the comparison of total Ade+ frequencies between the same strains with and without gRNA2, while in (B), they are relative to the equivalent wild-type strain value. The *p* values in (A) were calculated by the two-tailed Mann-Whitney test, while those in (B) were derived from the Kruskal-Wallis test with Dunn’s multiple comparisons post-test. ^∗∗∗∗^*p* < 0.0001; ^∗^*p* < 0.05; n.s., not significant. See also Table S1.

Template switching is a characteristic feature of BIR, which can be detected using a direct repeat reporter positioned downstream of the DSB from where BIR is initiated.^38^^,^^53^^,^^58^^,^^59^ Despite suspecting that lead- and lag-SSBs in *kan*^*R*^ are seldom converted into DSBs, we decided to investigate whether Cas9n-induced SSBs in *kan*^*R*^ can trigger BIR by measuring the frequency of Ade+ recombinants resulting from template switching at the adjacent *ade6*^*−*^ direct repeat reporter (Figure 3A). In the absence of gRNA2, expression of Cas9 or its mutant derivatives had no effect on the frequency of Ade+ recombinants (Figure 4A). We also saw no change in the frequency of Ade+ recombinants with expression of Cas9 and Cas9d in the presence of gRNA2 (Figure 4A). However, with Cas9n^H840A^ and Cas9n^D10A^, we observed marked increases in the frequency of both Ade+ gene conversions (5- and 18-fold, respectively) and Ade+ deletions (26- and 77-fold, respectively) (Figure 4A; Table S1). PCR analysis of a random sample of Ade+ His− recombinants confirmed that they were indeed deletions (Figure S3). Altogether, these data indicate that both lead- and lag-SSBs can induce BIR that drives template switching between direct repeats, which generates mainly deletions (Figure 4A). It takes a minimum of two template switch events to generate a gene conversion, whereas a deletion can be formed from a single template switch.^59^ This difference likely accounts for the deletion bias.

### Cas9n-induced BIR is promoted by Rad51, Pfh1, and Cdc27

To confirm that the Cas9n-induced Ade+ recombinants were from BIR-associated template switching, we tested their dependence on factors that are known to promote BIR (Figure 4B). Previous studies in budding yeast have shown that the vast majority of BIR in this organism depends on Rad51.^28^ However, Rad51-independent BIR has also been documented in yeast and mammalian cells,^28^ and in fission yeast, a related process termed recombination-dependent replication (RDR), that initiates when replication forks collapse at the *RTS1* replication fork barrier, can be driven without Rad51 by the DNA annealing activity of Rad52.^53^ A *rad52*-R45A mutant, which is defective for DNA annealing but proficient for mediating the loading of Rad51 onto RPA-coated ssDNA, exhibited wild-type levels of Cas9n-induced Ade+ recombinants (Figure 4B). By contrast, Cas9n-induced Ade+ recombinants were largely abolished in *rad51*Δ and *rad51*Δ *rad52*Δ mutants (Figure 4B). These data indicate that Cas9n-induced lead- and lag-SSBs in *kan*^*R*^ primarily give rise to Rad51-dependent BIR.

The Pif1 DNA helicase is known to be important for driving the elongation phase of BIR.^60^^,^^61^^,^^62^^,^^63^ As the Pif1 ortholog Pfh1 has an essential mitochondrial role in fission yeast, we used a *pfh1-mt^∗^* mutant that is specifically defective in nuclear localization to assess its requirement for Cas9n-induced template switching.^64^ The frequency of Ade+ recombinants induced by both Cas9n variants was reduced by ∼4-fold in a *pfh1-mt^∗^* mutant (Figure 4B). These data show that the formation of Cas9n-induced recombinants requires Pfh1, providing further evidence that they are derived from BIR-associated template switching. In budding yeast, BIR can progress at a diminished level for up to 5 kb from a DSB without Pif1.^62^ We suspect that this residual activity accounts for the remaining induced Ade+ recombinants in a *pfh1-mt^∗^* mutant that are particularly evident with lag-SSBs made by Cas9n^D10A^ (Figure 4B).

Another protein that plays an important role in the elongation phase of BIR in budding yeast and humans is the non-essential DNA polymerase ∂ subunit Pol32/POLD3.^14^^,^^32^^,^^62^^,^^65^ Unlike Pol32 and POLD3, their ortholog Cdc27 plays an essential role in DNA replication in fission yeast, so we used the cold-sensitive *cdc27-D1* mutant to assess its requirement for Cas9n-induced template switching.^66^^,^^67^ Similar to a *rad51*Δ *rad52*Δ mutant, Cas9n-induced Ade+ recombinants were largely abolished in a *cdc27-D1* mutant consistent with them arising from Cdc27-dependent BIR-associated template switching (Figure 4B).

### BIR is limited by fork convergence

**Figure 5:**
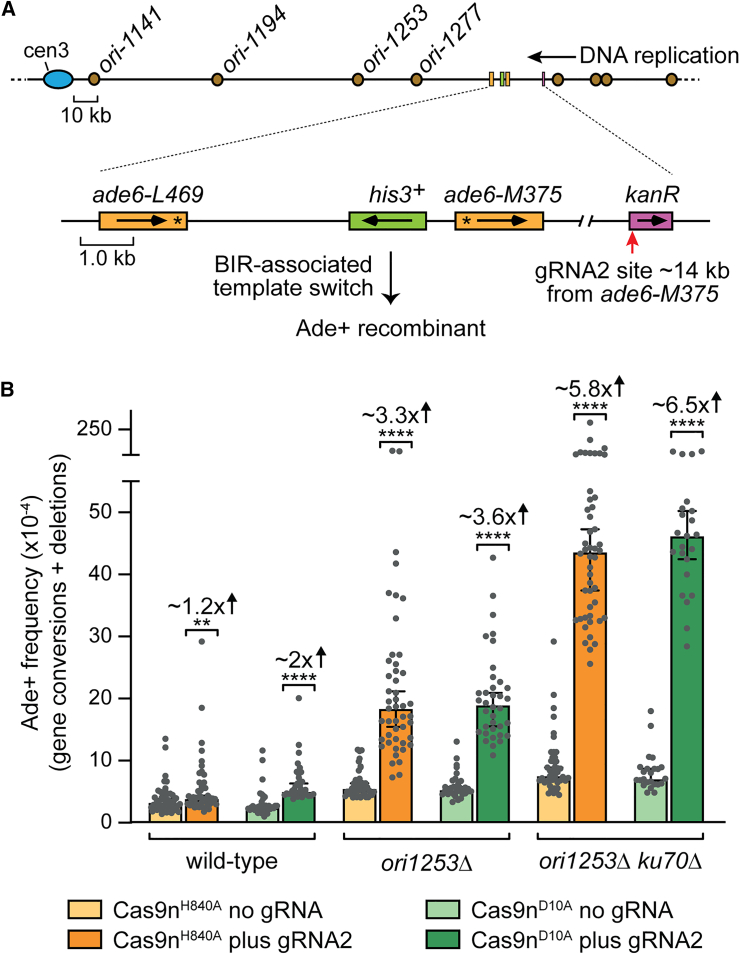
BIR is limited by fork convergence and suppressed by Ku70 (A) Diagram showing the location of *kan*^*R*^ gene (purple rectangle) and gRNA2 binding site (red arrow) ∼14 kb upstream of the same *ade6*^*−*^ direct repeat recombination reporter shown in Figure 3A. (B) Frequency of Cas9n-induced Ade+ recombinants in wild-type, *ori1253*Δ, and *ori1253*Δ *ku70*Δ strains containing the recombination reporter and *kan*^*R*^ gene shown in (A). The presence of gRNA2 and version of Cas9 is indicated. Data are presented as median values ± 95% confidence interval with individual data points shown as gray dots. The indicated fold changes and *p* values pertain to the comparison of recombination values between the same strains with and without gRNA2. The *p* values were calculated by the two-tailed Mann-Whitney test. ^∗∗∗∗^*p* < 0.0001; ^∗∗^*p* < 0.01. See also Table S1.

To investigate the extent of the BIR tract induced by Cas9n, we moved the *kan*^*R*^ gene ∼14 kb upstream of the *ade6*^*−*^ direct repeat reporter relative to the direction of replication (Figure 5A). In this scenario, we observed only ∼1.2-fold increase in the frequency of Ade+ recombinants with Cas9n^H840A^ and a ∼2-fold increase with Cas9n^D10A^ (Figure 5B). To see if this low level of template switching was a consequence of inefficient cleavage of *kan*^*R*^ at its new location, we examined genomic DNA extracted from asynchronously growing cell cultures for the presence of Cas9n-induced SSBs and DSBs by Southern blot analysis (Figure S4). Similar to the analysis of DNA breakage at the site between the *ade6*^*−*^ direct repeats (Figures 1B, 1C, and S1), we observed the presence of SSBs and deDSBs in *kan*^*R*^ with both Cas9n^H840A^ and Cas9n^D10A^ (Figure S4). However, the overall levels of DNA breakage in *kan*^*R*^ were noticeably lower than at the *ade6*^*−*^ direct repeat site by ∼4-fold.

Fork convergence is known to limit BIR induced from a site-specific SSB in *S. cerevisiae*^14^ to investigate whether the same holds true for lead- and lag-SSBs in *S. pombe*, we deleted the origin of replication *ori1253*, which is responsible for >70% of replication forks that converge on *kan*^*R*^ in the centromere to telomere direction.^38^^,^^68^ In an *ori1253*Δ mutant, there will be more time for BIR to initiate and progress to the downstream *ade6*^*−*^ direct repeat reporter as, in most cells, the converging replication fork now has to travel from one of the more distant centromere proximal origins (Figure 5A). In this scenario, both Cas9n variants induced a >3-fold increase in Ade+ recombinants in the presence of gRNA2 (Figure 5B). As this increase does not correlate with an increase in Cas9n-induced DNA breakage at *kan*^*R*^ (Figure S4), it most likely stems from BIR having sufficient time to progress to the downstream reporter in some cells. Altogether, these data indicate that both lead- and lag-SSBs can give rise to BIR, which can travel at least 14 kb from its initiating DNA lesion if not met by an opposing replication fork.

### Ku70 is a barrier to BIR

The Ku70-Ku80 (Ku) heterodimer has been implicated in binding reversed and broken replication forks, where it acts to limit DNA resection independently of its canonical role in NHEJ.^69^^,^^70^^,^^71^^,^^72^^,^^73^^,^^74^^,^^75^^,^^76^ To investigate whether Ku is a barrier to Cas9n-induced BIR, we assayed template switching in a *ku70*Δ *ori1253*Δ mutant using our reporter system (Figure 5A). In the presence of gRNA2, both versions of Cas9n induced higher levels of Ade+ recombinants in the *ku70*Δ *ori1253*Δ mutant than observed in the *ori1253*Δ single mutant (Figure 5B). These data indicate that Ku is a barrier to BIR induced by both lead- and lag-SSBs.

### Lead- and lag-SSBs induced by Flp^H305L^ and gpII trigger BIR

**Figure 6:**
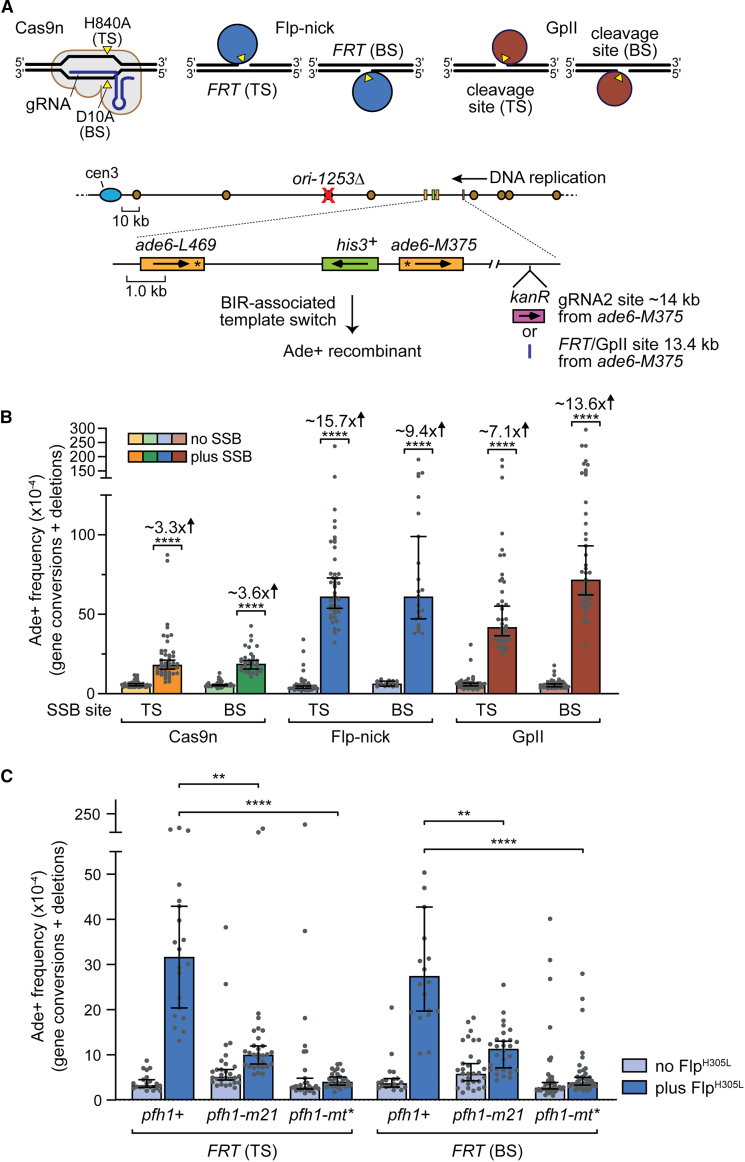
Lead- and lag-SSBs induced by Flp^H305L^ and gpII trigger BIR (A) Diagram showing the different site-specific-nicking enzymes used to generate lead- and lag-SSBs (top) and their cleavage sites located upstream of the *ade6*^*−*^ direct repeat recombination reporter on chromosome 3 (bottom). The recombination reporter and its location are the same as shown in Figures 3A and 5A. (B) Frequency of Ade+ recombinants induced by Cas9n, Flp-nick, and gpII in *ori1253*Δ strains containing the recombination reporter shown in (A). The position of the cleavage site in either the leading template (top) strand (TS) or lagging template (bottom) strand (BS) is indicated. For Cas9n, its expression without gRNA2 acts as a no SSB control. Absence of the nicking enzyme acts as the no SSB control for Flp-nick and gpII. (C) Frequency of Flp-nick-induced Ade+ recombinants in *pfh1*^+^*ori1253*Δ, *pfh1-m21 ori1253*Δ, and *pfh1-mt^∗^ ori1253*Δ strains containing the *ade6*^*−*^ direct repeat recombination reporter shown in (A). The data in (B) and (C) are presented as median values ± 95% confidence interval with individual data points shown as gray dots. The fold changes and *p* values in (B) refer to the comparison of recombination values between equivalent strains with and without SSB, with *p* values calculated using the two-tailed Mann-Whitney test. The *p* values in (C) relate to the comparison of recombination values with the equivalent *pfh1*^*+*^ strain and were calculated by the Kruskal-Wallis test with Dunn’s multiple comparisons post-test. ^∗∗∗∗^*p* < 0.0001; ^∗∗^*p* < 0.01. See also Table S1.

To investigate whether other types of SSB induce BIR, we used two sequence-specific-nicking enzymes, Flp^H305L^ and M13 bacteriophage gpII, to generate site-specific SSBs. Flp^H305L^ is a step-arrest mutant of the Flp recombinase that nicks DNA at Flp recognition target (*FRT*) sites, resulting in an SSB with Flp^H305L^ covalently linked to its 3′-phosphoryl terminus opposite a 5′-hydroxyl terminus (Figure 6A).^11^^,^^77^ These “Flp-nicks” mimic Top1ccs induced by CPT. By contrast, gpII binds covalently to the 5′ termini of the nicks it generates (Figure 6A).^10^^,^^12^^,^^78^ Importantly, both Flp-nick and gpII create SSBs that are converted into DSBs during DNA replication.^12^^,^^14^^,^^79^

We constructed strains with *FRT* or gpII cleavage sites located in either orientation ∼13.4 kb upstream of our recombination reporter relative to the direction of replication (Figure 6A). Since gpII nicks its cleavage site in a strand-selective manner, orientation of the site determines whether the SSB will be in the leading or lagging strand template (Figure 6A).^10^ Flp^H305L^ is also reported to have a strand cleavage bias *in vivo* (Figure 6A), but this bias is not observed *in vitro*, suggesting that it may generate similar levels of lead- and lag-SSBs regardless of *FRT* orientation.^11^^,^^14^^,^^77^^,^^80^ Both Flp^H305L^ and gpII significantly increased the frequency of Ade+ recombinants, ranging from a ∼7-fold increase for a lead-SSB made by gpII to a ∼15.7-fold increase for a Flp^H305L^ SSB with *FRT* in the “top strand” (TS) orientation (Figure 6B). Flp-nick-induced recombinants decreased in a *pfh1-m21* mutant with reduced nuclear Pfh1 levels and were almost abolished in a *pfh1-mt^∗^* mutant lacking nuclear Pfh1 (Figure 6C).^64^ This dependence on nuclear Pfh1 provides support that Flp-nick-induced recombinants arise from BIR-associated template switching.

Since the *FRT* and gpII cleavage sites were located at approximately the same site as the *kan*^*R*^ gene shown in Figure 5A, we could directly compare the frequencies of template switching induced by Cas9n, Flp-nick, and gpII in an *ori1253*Δ background (Figure 6B). Template switching with gpII and Flp-nick was ∼2–4 times higher than with Cas9n (Figure 6B). To see if this difference was due to gpII and Flp^H305L^ SSBs being generally more recombinogenic than those made by Cas9n, we compared recombination levels when their cleavage sites were located between the *ade6*^*−*^ heteroalleles of the direct repeat reporter (Figure S5). All three nicking enzymes induced similarly high levels of Ade+ recombinants in this context, indicating they are equally recombinogenic. Given that Cas9n-induced SSBs at *kan*^*R*^ with gRNA2 are ∼4-fold lower than at the site between the *ade6*^*−*^ direct repeats with gRNA1 (Figures S1 and S4), we suspect that the lower frequency of template switching with Cas9n, compared with gpII or Flp-nick (Figure 6B), is due to reduced nicking at *kan*^*R*^ rather than a fundamental difference in the ability of these SSBs to induce BIR.

## Discussion

**Figure 7:**
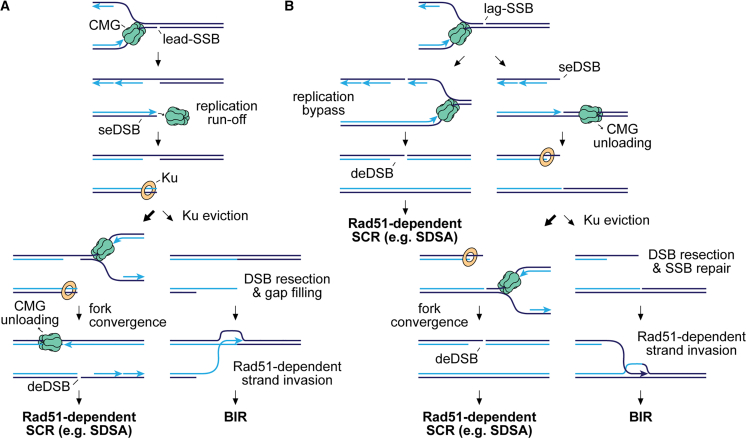
Model for the repair of DSBs that arise from replication fork encounters with SSBs (A) Diagram of replication run-off at a lead-SSB leading to the formation of a seDSB/deDSB and subsequent repair by SCR or BIR. (B) Diagram of replication bypass or CMG unloading at a lag-SSB leading to the formation of a seDSB/deDSB and subsequent repair by SCR or BIR. Parental DNA strands are shown in dark blue and nascent strands in light blue. Light and dark blue arrowheads indicate the direction of DNA synthesis.

In line with recent studies, our data indicate that encounters between replication forks and SSBs primarily give rise to deDSBs.^15^^,^^39^^,^^79^ Lead-SSBs are likely converted into deDSBs through a two-step process: first, CMG translocates off the DNA at the SSB, creating a seDSB, and then fork convergence at the SSB generates a deDSB (Figure 7A).^13^^,^^15^^,^^16^ By contrast, deDSBs at lag-SSBs can arise through at least three mechanisms, depending on the SSB’s nature: (1) replication bypass (Figure 7B),^15^^,^^39^ (2) CMG unloading leading to a seDSB, followed by fork convergence generating a deDSB (Figure 7B),^15^^,^^16^ and (3) fork stalling at Top1ccs followed by fork convergence.^81^ In all cases, the fork-SSB encounter results in one broken and one intact sister chromatid, creating an ideal scenario for HR to facilitate accurate DNA repair. Accordingly, our genetic data suggest that most of these deDSBs are repaired faithfully by Rad51-mediated SCR (Figure 7).^12^ However, the high frequency of gene conversions and deletions between *ade6*^*−*^ heteroalleles flanking the deDSB indicates that HR is not always accurate and can inadvertently recombine repetitive DNA sequences, as shown in previous studies (Figure S2A).^26^ Notably, we find that Rad52’s ssDNA annealing activity is required for some deletions formed in the presence of Rad51 (Figure 2B). Its role may be to promote the formation of a double Holliday junction through second end capture,^82^^,^^83^^,^^84^^,^^85^ or these deletions might arise from Rad52-mediated SSA, which is known to compete with Rad51-dependent DSB repair.^41^^,^^46^^,^^86^

Even though the majority of fork-SSB encounters appear to be resolved through the formation and repair of a deDSB, our data indicate that some lead to BIR. Since BIR is rarely observed at deDSBs, we suspect that most of the BIR events we detect originate from seDSBs.^26^^,^^87^ Importantly, we show that both lead- and lag-SSBs are capable of triggering BIR, implying that replication fork encounters with both types of SSB can generate seDSBs *in vivo* (Figure 7). This finding is consistent with *in vitro* analyses of replication fork breakage at lead- and lag-SSBs induced by Cas9n^H840A^.^16^ However, it seems to contradict the observation that lag-SSBs induced by Cas9n^D10A^ predominantly lead to deDSBs.^15^ It has been suggested that the nature of the lag-SSB may influence whether the replication fork bypasses it, with lag-SSBs generated by Cas9n^D10A^ and Flp-nick being readily bypassed, while those induced by Cas9n^H840A^ are not.^15^^,^^79^ While CMG may bypass certain types of lag-SSBs more easily than others, our observation that BIR-associated template switching can be triggered by a lag-SSB created by Cas9n^D10A^ suggests that even nicks capable of being bypassed are not always successfully navigated. Indeed, END-seq analysis of DSBs formed at lag-SSBs made by Cas9n^D10A^ in human cells revealed some asymmetry in the amount of DSB signal on either side of the Cas9n^D10A^ cleavage site, suggesting that a minority of DSBs were single-ended.^15^

Considering that a replication fork encountering a lead-SSB is more likely to generate a seDSB than one encountering a lag-SSB created by Cas9n^D10A^, it is surprising that we see similar levels of template switching induced by both lead- and lag-SSBs. However, this discrepancy may be explained by differences in the nature of the seDSBs, which could affect how far BIR can progress before being terminated by an oncoming replication fork. Replication run-off at a lead-SSB (lead-collapse) generates an almost blunt seDSB plus an unbroken sister chromatid with a ssDNA gap between the final primed Okazaki fragment and the 3′ terminus of the SSB (Figure 7A).^16^ By contrast, CMG disassembly following encounter with a lag-SSB (lag-collapse) generates a seDSB with a ∼70 nucleotide 3′ ssDNA overhang plus an unbroken sister chromatid with just a nick (Figure 7B).^16^ It has been suggested that these differences could have an impact on the subsequent repair. Repair of lead-collapse seDSBs might be more reliant on gap filling and DNA end resection, whereas lag-collapse seDSBs would require no gap filling and have a ready-made 3′ ssDNA tail sufficient for Rad51-mediated strand invasion.^16^ Such differences could also have an impact on the speed at which BIR is initiated, with the additional processing requirements of lead-collapsed forks resulting in slower BIR initiation. Therefore, although lag-SSBs may produce fewer seDSBs compared with lead-SSBs, faster initiation of BIR could result in longer BIR tracts on average and a similar overall level of template switching induction at our genetic reporter located ∼14 kb downstream of the Cas9n cleavage site.

The frequency of BIR-associated template switching increases in a *ku70*Δ mutant, indicating that Ku suppresses BIR (Figure 5B). If BIR is initiated from deDSBs that can be repaired by NHEJ, then loss of Ku could conceivably channel more repair through BIR. However, we prefer the idea that Ku acts as a barrier to BIR by binding to the seDSB, thereby delaying the recruitment of HR proteins (Figure 7). The difference in structure of lead- and lag-collapse seDSBs has led to speculation that Ku might have a bigger influence on the processing of the former than the latter.^16^ However, we observe an almost identical increase in BIR-associated templating switching with both lead- and lag-SSBs when *ku70* is deleted (Figure 5B). This suggests that Ku is a barrier to BIR regardless of whether it is initiated from a lead- or lag-collapse. It should be noted that, while Ku binds less efficiently to ssDNA than dsDNA, it can localize to ssDNA-dsDNA junctions and protect 5′-recessed ends from degradation by Exo1 *in vitro*.^88^

In budding yeast outside of S phase, BIR is a slow process, with the first 500 bp of DNA synthesis detected 2.5 h after DSB induction.^62^ Once initiated, the average rate of BIR DNA synthesis is 0.5 kb per min, which is 4 times slower than a typical replication fork in *S. cerevisiae*.^62^^,^^89^ This slow kinetics likely limits BIR’s effectiveness in repairing broken replication forks, as fork convergence usually occurs before BIR can begin. In our system, template switching ∼14 kb downstream of the SSB was only clearly detected when fork convergence was delayed by deleting *ori1253*. In this case, the converging fork mainly originates from either *ori1194* or *ori1141* (Figure 5A). If at least one of these origins fires during early S phase, the resulting fork, moving at an average velocity of 2 kb per min, would allow BIR less than 1.5 h to register a template switch at the downstream reporter.^38^ This suggests that either BIR operates faster from a broken replication fork in *S. pombe* than from a non-S phase DSB in *S. cerevisiae*, or the template switches we detect only occur in cells where the converging fork experiences additional delays.

Our finding that different types of lead- and lag-SSBs are able to give rise to BIR-associated template switching suggests that this is a universal response to fork-SSB encounters. However, our data and other recent studies also indicate that these encounters are more frequently resolved through the formation and repair of deDSBs.^15^^,^^39^^,^^79^ This provides a safer route for maintaining genome stability as more faithful repair pathways such as SDSA can be used. Timely fork convergence appears to be a critical factor in ensuring that seDSBs are converted into deDSBs. The presence of Ku at seDSBs may also help to delay BIR, providing more time for fork convergence to occur. In future studies, it will be important to determine whether there are other factors that govern the deployment of BIR to ensure that this mutagenic mode of DNA synthesis is only unleashed as a pathway of last resort.

### Limitations of the study

Assessing the frequency of template switching at a direct repeat reporter provides an indirect measure of BIR. While we can infer from our data that BIR is occurring and gain an indication of its relative frequency in different genetic backgrounds, its rate of deployment per fork-SSB encounter remains undetermined.

## Resource availability

### Lead contact

Further information and requests for resources and reagents should be directed to and will be fulfilled by the lead contact, Matthew Whitby (matthew.whitby@bioch.ox.ac.uk).

### Materials availability

All yeast strains and DNA constructs generated in this study will be made available upon request.

### Data and code availability

• Uncropped Southern blot images and recombination raw data have been deposited at Mendeley and are publicly available as of the date of publication. The DOI is listed in the key resources table.
• The data do not report original code.
• Any additional information required to reanalyze the data reported in this paper is available from the lead contact upon request.

## Acknowledgments

We thank Lotte Bjergbæk, Ian Hagan, and Timothy Humphrey for the gift of strains/plasmids. This study was supported by grants from the Medical Research Council (MR/V009214/1) and Biotechnology and Biological Sciences Research Council (BB/P019706/1 and BB/V00073X/1). The funders had no role in study design, data collection, and analysis; the decision to publish; or preparation of the manuscript.

## Author contributions

Conceptualization, Y.X., C.A.M., and M.C.W.; methodology, Y.X., C.A.M., Y.L., and M.C.W.; investigation, Y.X., C.A.M., Y.L., O.M.H., K.T., C.T., and P.C.; writing – original draft, M.C.W.; writing – review and editing, Y.X., C.A.M., and M.C.W.; supervision, Y.X., C.A.M., C.A., and M.C.W.; and funding acquisition, M.C.W.

## Declaration of interests

The authors declare no competing interests.

## Declaration of generative AI and AI-assisted technologies in the writing process

During the final editing of this article, the authors used ChatGPT to improve the text’s conciseness. After using this tool, the authors reviewed and edited the content as needed and take full responsibility for the content of the publication.

## STAR★Methods

### Key resources table

**Table:** 

REAGENT or RESOURCE	SOURCE	IDENTIFIER
**Chemicals, peptides, and recombinant proteins**		

G418 disulfate salt	Sigma-Aldrich	A1720
Taq polymerase + ThermoPol® Reaction Buffer	NEB	M0267L
3-BrB-PP1	Abcam	ab143756
Calcofluor White Stain	Sigma-Aldrich	18909
*Trichoderma harzianum* lysing enzymes	Sigma-Aldrich	L1412
*Arthrobacter luteus* lyticase	Sigma-Aldrich	L4025
Zymolyase 20T	MP Biomedicals	08320922
DTT	Sigma-Aldrich	D9779
low-melting point agarose	Thermo Scientific	R0801
rCutSmart buffer	NEB	B6004S
Beta-Agarase I	NEB	M0392
PstI-HF	NEB	R3140L
NheI-HF	NEB	R3131L
XmnI	NEB	R0194L
RNase A	Qiagen	1007885
phenol:chloroform:isoamyl alcohol	ThermoFisher Scientific	15593031
Rediprime II Random Prime Labelling System	Cytiva	RPN1633
alpha-^32^P dCTP	Revvity	NEG513H250UC
ULTRA-hyb buffer	Invitrogen	AM8669

**Deposited data**		

Raw and analyzed data	This paper	Mendeley Data: https://doi.org/10.17632/phgpwmjvys.1

**Experimental models: Organisms/strains**		

*S. pombe* strains used in this study are listed in Table S3	This paper	N/A

**Oligonucleotides**		

Oligonucleotides used in this study are listed in Table S4	This paper	N/A

**Recombinant DNA**		

Plasmid: pLX27	This paper	N/A
Plasmid: pLX1	This paper	N/A
Plasmid: pLX5	This paper	N/A
Plasmid: pLX6	This paper	N/A
Plasmid: pLX10	This paper	N/A
Plasmid: pLX2	This paper	N/A
Plasmid: pLX3	This paper	N/A
Plasmid: pLX32	This paper	N/A
Plasmid: pLX46	This paper	N/A
Plasmid: pLX47	This paper	N/A
Plasmid: pLX44	This paper	N/A
Plasmid: pYL8	This paper	N/A
Plasmid: pYL9	This paper	N/A
Plasmid: pKT1	This paper	N/A
Plasmid: pKT2	This paper	N/A
Plasmid: pYL7	This paper	N/A
Plasmid: pYL4	This paper	N/A
Plasmid: pREP1	Maundrell^90^	N/A
Plasmid: pPC7	This paper	N/A
Plasmid: pREP1-NLS-gpII	Osman et al.^12^	N/A
Plasmid: pOMH1	This paper	N/A
Plasmid: pOMH2	This paper	N/A

**Software and algorithms**		

Science Lab 2001, Image Gauge Version 4.21	Fujifilm	N/A
Excel for Mac Version 16.78.3	Microsoft®	http://www.microsoft.com
GraphPad Prism Version 10.1.0	GraphPad Software, San Diego, CA	http://www.graphpad.com

**Other**		

Nalgene Rapid-Flow filters	Thermo Scientific	597-4520
CHEF disposable plug molds	BioRad	1703713
DNA LoBind tubes	Eppendorf	1130122232; 0030122208; 022431021
GeneScreen Plus membrane	Revvity	NEF988001PK

### Experimental model and study participant details

The *S. pombe* strains used in this study are derivatives of the heterothallic strains 972 h- and 975 h+.^91^ They were grown by plating samples from frozen stocks onto yeast media and incubating at 25°C or 30°C.

### Method details

#### Yeast strains and plasmid construction

*S. pombe* strains and oligonucleotides are listed in Tables S3 and S4, respectively. Derivatives of recombination reporter strains carrying the indicated gene/replication origin deletion(s) were obtained from genetic crosses. Strain MCW9804 was constructed by transformation of BlpI-linearized plasmid pLX27 into a *ade6-M375* strain.^92^ pLX27 is a derivative of pCB33 in which the *RTS1* sequence has been removed by SacI digestion.^93^ Plasmids pLX1, pLX5, pLX6 and pLX10 are derivatives of pAG32,^94^ which were used for the targeted integration of P*nmt81*-*cas9*/*cas9n*/*cas9d*-*hphMX4* at the *lys1* locus. pLX1 contains *cas9n*^*D10A*^, which was cloned from pSPCas9n(BB)-2A-GFP (Addgene plasmid ID: #48140).^95^ pLX5 (*cas9d*^*D10A/H840A*^), pLX6 (*cas9n*^*H840A*^), and pLX10 (*cas9*), were derived from pLX1 by site-directed mutagenesis of *cas9n*^*D10A*^ to obtain the required version of Cas9. All versions of Cas9 were expressed from the thiamine repressible *nmt81* promoter.^96^ Plasmid pLX2 is a derivative of pMZ374 (Addgene plasmid ID: #59896)^97^ in which the Cas9 gene has been removed by EagI digestion, and annealed oligonucleotides, oMW1708 plus oMW1709, inserted at the CspCI site. This plasmid contains a *ura4*^*+*^ marker and expresses gRNA1 that anneals to the lagging strand template in the region adjacent to the 5' end of the *his3* gene in the *ade6*^*-*^ direct repeat recombination reporter shown in Figures 1A and S5A. pLX3 is a derivative of pLX2 that lacks the sequence for the gRNA. It was constructed by digesting pLX2 with BamHI and NheI to remove the gRNA sequence, and then re-ligating the DNA ends after blunt ending them with DNA Polymerase I, Large (Klenow) Fragment (New England BioLabs, M0210L). pLX32 is a derivative of pMZ283 (Addgene plasmid ID: #52224),^97^ which was obtained by site-directed mutagenesis of pMZ283 using primers oMW2158 and oMW2159. This plasmid contains a *ura4*^*+*^ marker and expresses gRNA2, which anneals to the lagging strand template at the 5′ end of the *kan*^*R*^ gene shown in Figures 3A, 5A, and 6A. pLX46 and pLX47 are derivatives of pLX32 and pMZ283, respectively, which contain a *LEU2* gene instead of *ura4*. The construction of the *ade6*^*-*^ direct repeat used to monitor template switching has been described previously.^53^^,^^59^ Placement of the *kan*^*R*^ gene at the *ade6* locus, ∼14 kb upstream of this reporter, was done by targeted gene replacement using a NotI restriction fragment from pLX44. pLX44 was derived from pMW921^38^ by swapping a SalI-SacI fragment containing *RTS1*-*hphMX4* for the *kanMX6* cassette from pFA6a-KanMX6.^98^ Placement of *FRT* sites and gpII cleavage sites upstream of the template switch reporter was also done by targeted gene replacement of *ade6* using NotI restriction fragments from plasmids pYL8 (gpII TS cleavage site), pYL9 (gpII BS cleavage site), pKT1 (*FRT* BS cleavage site), and pKT2 (*FRT* TS cleavage site). These plasmids were made by swapping a SalI-SmaI fragment in pMW921, containing *RTS1*, for the following annealed oligonucleotides: oMW480 and oMW481 (pYL8); oMW482 and oMW483 (pYL9); oMW2031 and oMW2032 (pKT1); oMW2068 and oMW2069 (pKT2). Strains MCW10107 and MCW10108, which contain the *ade6*^*-*^ direct repeat recombination reporter with an *FRT* site between *his3* and *ade6-M375*, were made by transformation of BlpI-linearised plasmid pYL7 and pYL4, respectively, into a *ade6-M375* strain. Both plasmids are derivatives of pFOX2^92^ with annealed oligonucleotides, oMW2031 plus oMW2032 (pYL7) or oMW2068 plus oMW2069 (pYL4), inserted between SalI and SmaI restriction sites. The plasmids for expression of Flp^H305L^ and gpII are derivatives of pREP1.^90^^,^^99^ pREP1-Flp^H305L^ (pPC7) was made by first amplifying the Flp^H305L^ gene from plasmid pFV17D-H305L^11^ using primers oMW2029 and oMW2030, and then cloning it as a SalI-BamHI fragment into pREP1 so that its expression would be directed by the thiamine repressible *nmt1* promoter. pOMH1 and pOMH2 are derivatives of pREP1 and pPC7, respectively, that contain an *arg3*^+^ marker in place of *LEU2*. The construction of pREP1-NLS-gpII has been described.^12^ Plasmids were verified by DNA sequencing. The genotype of each *S. pombe* strain was confirmed by determining the presence/absence of relevant genetic markers, as well as diagnostic PCRs and DNA sequencing where appropriate.

#### Media and growth conditions

Standard protocols were used for the growth and genetic manipulation of *S. pombe*.^100^ The complete and minimal media were yeast extract with supplements (YES) and Edinburgh minimal medium plus 3.7 g/l sodium glutamate (EMMG) and appropriate amino acids (225 mg/l), respectively. Strains carrying the integrated P*nmt81*-*cas9/cas9n/cas9d* constructs or pREP1/pREP1-FlpH305L/gpII were grown on EMMG supplemented with thiamine (24 μM final concentration) to repress expression from the *nmt* promoter. Strains carrying plasmids were grown on EMMG lacking leucine or uracil as appropriate. To maintain the integrity of the *ade6-L469* – *ade6-M375* recombination reporters, strains carrying them were grown on EMMG lacking histidine and supplemented with low levels of adenine (10 mg/l) to distinguish non-recombinant colonies (red) from Ade+ recombinants (white). For recombination assays, the total number of colony forming cells was determined by plating onto YES supplemented with low levels of adenine (10 mg/l) (YES/LA). Ade+ recombinants were selected on YES lacking adenine and supplemented with 200 mg/l of guanine (YES-ade+gua) to prevent uptake of residual adenine.^101^ All strains were grown at 30°C, unless otherwise stated.

#### Spot assay

Cells were cultured in EMMG medium lacking uracil, with or without thiamine, at 30˚C until they reached mid-exponential phase. Following this, the cells were harvested, washed with sterile Milli-Q water, and counted using a haemocytometer. The cells were then resuspended in sterile Milli-Q water to a density of 1 x 10^7^ cells per ml and serially diluted in tenfold increments to 1 x 10^4^ cells per ml. Aliquots of 10 μl from each dilution were spotted onto EMMG agar plates, also lacking uracil and supplemented with or without thiamine. The plates were incubated at 30°C for 5 days, after which they were photographed.

#### G418 sensitivity assay

Relevant strains carrying the *kan*^*R*^ gene adjacent to the *ade6*^*-*^ direct repeat recombination reporter on chromosome 3 (Figure 3A), were transformed with the gRNA2 expressing plasmid pLX32 or the no gRNA control plasmid pLX3. Five independent transformants for each strain were plated onto EMMG agar lacking uracil and thiamine. Colonies were grown for 6 days at 30°C. For the data in Figures 3E and 3F, the colonies that had grown on EMMG agar lacking uracil and thiamine were directly replica plated onto YES and YES containing G418 (100 mg/l, Sigma-Aldrich, A1720), and then incubated for 24 hours at 30°C before being photographed and counted. For the data in Table S2, one colony from each of the five independent transformants, that had been grown on EMMG agar lacking uracil and thiamine for 6 days, was suspended in sterile Milli-Q water, serially diluted, and plated onto YES. These plates were then incubated for 3 days at 30°C before being replica plated onto YES and YES containing G418 (100 mg/l). The replica plates were then incubated for 24 hours at 30°C before being photographed and their colonies counted.

#### Recombination assays

The protocol for determining the frequency of Ade^+^ recombinants has been described.^101^ Strains transformed with the appropriate gRNA/Flp^H305L^/gpII/control plasmid were grown for 6 - 10 days on EMMG plates lacking either uracil or leucine for plasmid selection, and also lacking thiamine to allow for Cas9/Cas9n/Cas9d/Flp^H305L^/gpII expression and the induction of recombination. Colonies from these plates were then analysed for their frequency of Ade^+^ recombinant cells. This was done by serially diluting each colony in sterile Milli-Q water and then plating appropriate dilutions on YES/LA and YES-ade+gua. These plates were grown for 5 - 6 days. Colonies were then counted using a Protos 3 automated colony counter (Synbiosis) with Protos 3 Version 1.2.4.0 software (Synoptics Ltd). The frequency of Ade+ recombinants amongst total viable (colony forming) cells was determined by comparing the number of colonies on the YES/LA plate with those on the YES-ade+gua plate. The percentage of deletions and gene conversions among Ade+ recombinants was determined by replica plating the YES-ade+gua plates onto EMMG plates that lacked histidine. For each strain, at least three independent gRNA/Flp^H305L^/gpII/control plasmid transformants were assayed and, for each of these transformants, the recombination frequency was measured in 4 - 15 separate colonies. A single recombination event at an early stage in the growth of a colony will give rise to many recombinant cells. To avoid these so-called jackpot events from skewing the data, we used the method of the median to determine the recombination frequency for each strain.

#### Colony PCR analysis of Ade+ recombinants

Random Ade+ His+ and Ade+ His- colonies from recombination assay YES-ade+gua plates were selected and used directly as templates for colony PCR. Each individual PCR mix contained 1x ThermoPol® Reaction Buffer (New England BioLabs, M0267L), 200μM dNTPs, 200 nM of each primer (oMW285 and oMW1617), 0.94 units Taq polymerase (New England BioLabs, M0267L), and the Ade+ yeast colony as the template, in a total volume of 30 μl. The cycling conditions were as follows: 1 cycle of 98°C for 5 minutes; 35 cycles of 95°C for 15 seconds, 50°C for 30 seconds, 68°C for 10 minutes; followed by 1 cycle at 68°C for 10 minutes.

#### Cell culture for DSB/SSB detection

For analysis of DSB/SSB in asynchronously growing cell cultures, strains were grown in shake flasks containing 500 ml EMMG (lacking histidine, uracil and thiamine in the case of Figure 1B; or leucine and thiamine in the case of Figure S4) for 48 - 50 hours reaching a density of ∼1x10^7^ cells/ml. The cultures were then treated with 0.1% sodium azide and harvested by centrifugation. Cell pellets were washed in 20 ml 50 mM EDTA and stored at -80°C.

To analyse DSB/SSB in synchronously growing cell cultures, strains containing the *cdc2-asM17* allele,^102^ Cas9n^H840A^ variant and gRNA1 plasmid were initially grown overnight at 30°C in starter cultures of EMMG lacking histidine and uracil, supplemented with 24 μM thiamine to suppress gRNA expression. The following day, cells were washed and diluted into 500 ml of fresh EMMG media without thiamine. After 23 hours, the cultures were washed three times in media and resuspended in 500 ml of fresh media at a concentration of 1 x 10^7^ cells/ml at 25°C for one hour. The ATP analogue 3-BrB-PP1 (Abcam, ab143756) was then added to a final concentration of 5 μM, and the cells were incubated for an additional 4 hours and 15 minutes at 25°C. The G2-M synchronised cultures were subsequently filtered using 1000 ml, 0.2 μm pore size, 90 mm diameter Nalgene Rapid-Flow filters (Thermo Scientific, 597-4520), washed three times with excess pre-warmed media to remove drug, and resuspended in fresh media to release the cells into mitosis. Cultures were allowed to grow for an additional two hours, with samples taken every half hour. These samples were treated and stored as described for asynchronously growing cell cultures. At each time point, separate 1 ml samples were also harvested, centrifuged, and resuspended in 1 ml of ice-cold 70% ethanol to fix the cells. 2 μl of each fixed sample was mixed with 2 μl of Calcofluor White Stain (1 g/l, Sigma-Aldrich, 18909) and examined for septation, which indicates progression into mitosis, using an epifluorescence microscope with a DAPI filter (excitation 358 nm, emission 460 nm). At least 300 cells were scored for each time point.

#### DSB and SSB detection

Frozen cells were thawed on ice and gently resuspended in an equal volume of CPES buffer (40 mM citric acid, 120 mM Na_2_PO_4_, 400 mM EDTA, 1.2 M sorbitol). To this mixture, *Trichoderma harzianum* lysing enzymes (250 mg/ml; Sigma-Aldrich, L1412), *Arthrobacter luteus* lyticase (25 mg/ml; Sigma-Aldrich, L4025), Zymolyase 20T (100 mg/ml; MP Biomedicals, 08320922), and DTT (10 μl/ml of a 1 M solution; Sigma-Aldrich, D9779) were added. The suspension was incubated at 37°C for up to 2 hours until spheroplasting was achieved. The spheroplasted cells were then mixed with an equal volume of molten 2% low-melting point agarose (Thermo Scientific, R0801) in 0.25 M EDTA and 1.2 M sorbitol. The cell/agarose mixture was pipetted into plug molds (BioRad, 1703713) and allowed to set at 4°C for 15 minutes. Plugs were subsequently removed from the molds and incubated for 36 hours at 50°C in 3 ml of NDS-PK buffer (10 mM Tris-HCl pH 7.5, 1% N-lauroylsarcosine, 495 mM EDTA, 0.5 mg/ml Proteinase K), with the buffer replaced with a fresh 3 ml aliquot after 18 hours. Following incubation, the plugs were washed for 1 hour in 10 ml of 1x TE buffer (10 mM Tris-HCl pH 8.0, 1 mM EDTA) containing 0.5 mM PMSF, followed by three 1-hour washes in 20 ml of 1x TE. The plugs were then stored overnight at 4°C in 1x TE.

For all subsequent steps, DNA LoBind tubes (Eppendorf: 1130122232; 0030122208; 022431021) were used. The plugs were washed in 20 ml of Milli-Q water for 30 minutes and then in 10 ml of 1x rCutSmart buffer (New England Biolabs, B6004S) for 1 hour, followed by an additional hour in 5 ml of 1x rCutSmart buffer. After removing the buffer, the plugs were melted by placing the tube in a 65°C water bath for 15 - 20 minutes. The tube was then cooled to 42°C before adding 5 μl of Beta-Agarase I (New England Biolabs, M0392) and incubating at 42°C for 1 hour. Next, 200 units each of PstI-HF (New England Biolabs, R3140L) and NheI-HF (New England Biolabs, R3131L) (Figures 1 and S1) or 200 units XmnI (New England Biolabs, R0194L) (Figure S4), along with 20 μl of RNase A (100 mg/ml; Qiagen; 1007885), were added. The mixture was incubated overnight at 37°C. An additional 100 units of PstI-HF and NheI-HF (Figures 1 and S1), or 100 units XmnI (Figure S4), were added the following day, and incubation continued for another 6 hours. The mixture was then centrifuged at 3,200 x g for 10 minutes, and the supernatant was transferred to a fresh tube. The supernatant was mixed with an equal volume of phenol:chloroform:isoamyl alcohol (25:24:1 v/v; ThermoFisher Scientific, 15593031) and centrifuged for 10 minutes. DNA in the aqueous phase was precipitated by adding an equal volume of isopropanol plus 0.1 volume of 3 M sodium acetate and incubating overnight at -20°C. After centrifugation, the DNA pellet was washed three times with 70% ethanol, dried, and dissolved in either 40 μl (DSB detection only) or 80 μl (both SSB and DSB detection) of 1x TE buffer.

For DSB detection, 40 μl samples were mixed with 4 μl of 10 x gel loading dye (50% glycerol, 1% SDS, 0.1 M EDTA, 0.25% bromophenol blue), and equal-sized aliquots (∼10 μg of DNA in each) were run on separate 1% agarose gels in 1x TBE buffer for 24 hours at 50 V. Gels were stained with Ethidium Bromide for 1 hour to visualise the DNA before Southern blotting. For SSB detection, 40 μl samples were mixed with 0.8 μl 0.5 M EDTA (pH 8.0) and 7 μl of 6x alkaline gel loading buffer (50 mM NaOH, 1 mM EDTA, 3% Ficoll type 400, 2.5% bromocresol green, 4% xylene cyanol). Samples were split into equal aliquots and run on 1% agarose gels prepared in freshly made 1x alkaline gel electrophoresis buffer (50 mM NaOH, 1 mM EDTA pH 8.0). Alkaline gels were run at 3.5 V/cm in 1x alkaline gel electrophoresis buffer, stained with Ethidium Bromide, and neutralised in 1 M Tris (pH7.6)/1.5 M NaCl, before Southern blotting.

For Southern blotting, gels were treated with 0.25 M HCl to depurinate the DNA before transfer to GeneScreen Plus membrane (Revvity, NEF988001PK) by capillary action under alkaline conditions. Following transfer, membranes were probed with either ^32^P-radiolabelled probe A, S, D or F (Rediprime II Random Prime Labelling System, Cytiva, RPN1633; and alpha-^32^P dCTP, Revvity, NEG513H250UC) in ULTRA-hyb buffer (Invitrogen, AM8669) at 42°C. The membrane was then washed following the manufacturer's recommended procedure and exposed to a phosphor screen for up to 5 days. The phosphor screen was scanned using a FLA-3000 Phosphorimager (Fujifilm) controlled by Image Reader software (Fujifilm, Version 2.02).

#### DNA probes

The DNA templates for random prime labelling of probes were as follows: Probe A is a 1047 bp EcoNI + XbaI restriction fragment from pFOX2, representing the majority of the *his3* gene; Probe S is a 1330 bp SacI + Eag1 restriction fragment from pLX27; Probe D is a 718 bp fragment of the *bub1* gene, amplified from *S. pombe* genomic DNA using primers oMW706 and oMW707; Probe F is a 702 bp DNA fragment of the *vtc4* gene, amplified from *S. pombe* genomic DNA using primers oMW705 and oMW1627.

### Quantification and statistical analysis

The quantification of DSB signals, visualised by phosphorimaging of Southern blots, was performed using Image Gauge software (Fujifilm, Version 4.21). The DSB signal is expressed as a percentage of the total DNA signal (unbroken DNA + DSB signal). To account for the contribution of both nicked and un-nicked DNA to the unbroken DNA band observed on denaturing gels, the percentage of nicked dsDNA molecules was calculated by multiplying the % SSB signal by two. For the data in Figures 1C and S4, background signals were corrected by subtracting the percentage DSB/SSB signal from the minus gRNA sample from that of the +gRNA sample. In Figure S1, SSB/DSB percentages were calculated after subtracting the local background signal from both unbroken and SSB/DSB signals. The data are presented as mean values ± SD from three biological replicates, except for the data in Figure S1, which are from two biological replicates and presented as mean values ± SEM.

Recombination frequencies were obtained by analysing at least eleven colonies across at least three independent transformants, and are expressed as median values ± 95% confidence interval. Data analysis was conducted using Excel for Mac Version 16.78.3 (Microsoft®) and GraphPad Prism Version 10.1.0 (GraphPad Software, San Diego, CA). Recombination frequencies were assessed for normal distribution using the Shapiro-Wilk test. As not all datasets passed this test, we subsequently evaluated them for lognormal distribution. Data conforming to a lognormal distribution were log-transformed, and relevant recombination frequencies were compared using an unpaired two-tailed t-test. For datasets that did not follow a lognormal distribution, comparisons were made using a two-tailed Mann-Whitney test or a Kruskal-Wallis test (one-way ANOVA on ranks) followed by Dunn’s multiple comparisons post-test. These non-parametric statistical tests do not require the data to be normally distributed. The *p* values are reported in Tables S1 and S5.

## Supplemental information

Document S1. Figures S1–S5, Tables S2 and S4, and supplemental references

Table S1. Recombination frequencies, related to Figures 1, 2, 4, 5, 6, and S5

Table S3. List of *S. pombe* strains, related to STAR Methods

Table S5. Details of statistical analysis, related to STAR Methods

Document S2. Article plus supplemental information
