# Supplementary material for: DNA nicks in both leading and lagging strand templates can trigger break-induced replication
Source: Mol Cell. 2025 Jan 2;85(1):91–106.e5. doi: 10.1016/j.molcel.2024.10.026 (PMC12095120; doi:10.1016/j.molcel.2024.10.026)
Supplement: Document S1. Figures S1–S5, Tables S2 and S4, and supplemental references [file mmc1.pdf]

Molecular Cell, Volume *85*

Supplemental information

DNA nicks in both leading and lagging strand templates can trigger break-induced replication

Yuanlin Xu, Carl A. Morrow, Yassine Laksir, Orla M. Holt, Kezia Taylor, Costas Tsiappourdhi, Patrick Collins, Su Jia, Christos Andreadis, and Matthew C. Whitby

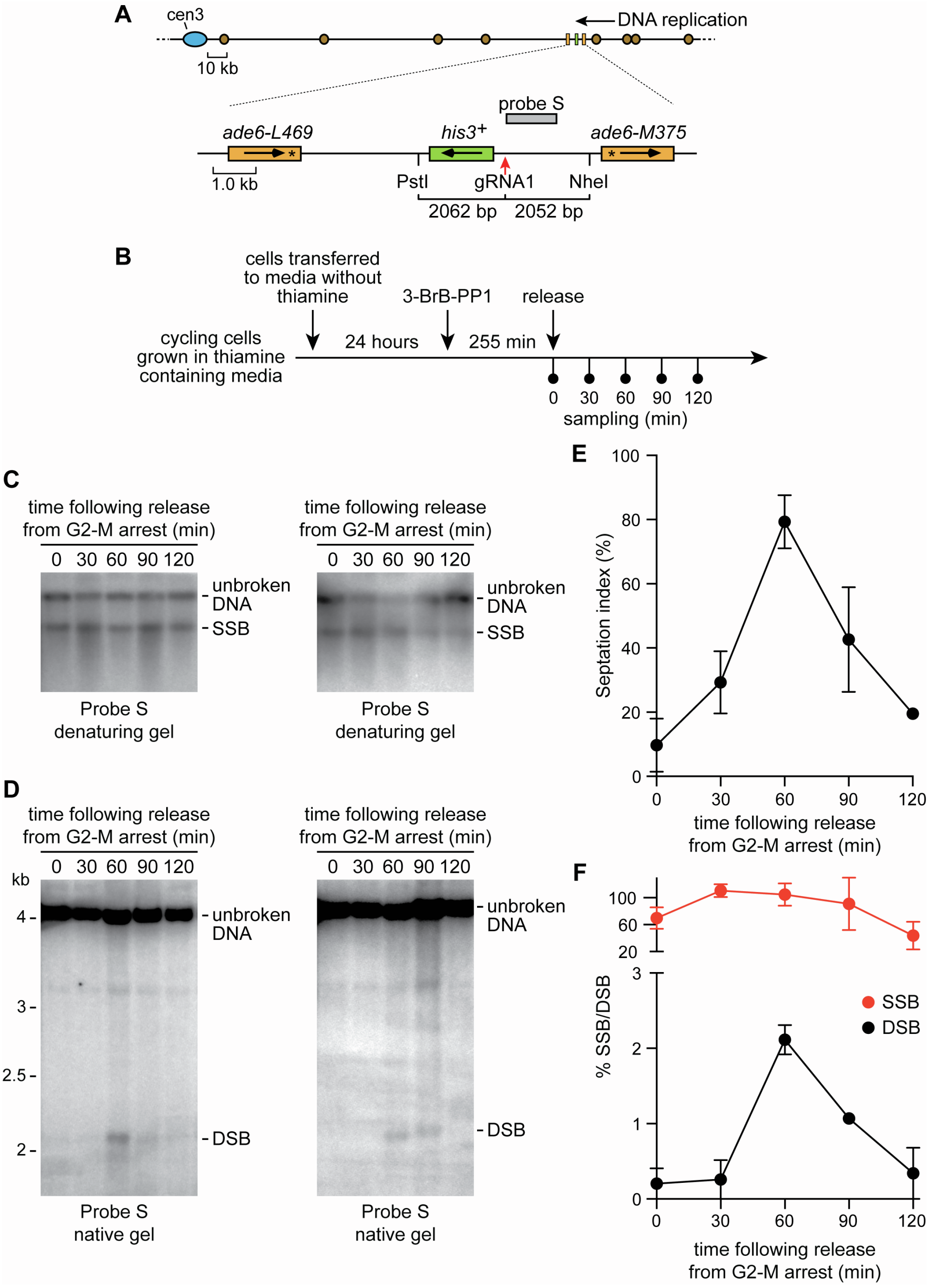

Figure S1. Cas9n^{H840A}-induced SSBs are converted into DSBs during S-phase, related to Figure 1.

(A) Diagram showing the location of the gRNA1 binding site (red arrow) on chromosome 3. Centromere (blue oval), replication origins (brown circles), probe (grey rectangle), relevant restriction sites, and genes (orange and green rectangles with arrows indicating the direction of transcription) are depicted.

(B) Schedule for: de-repressing Cas9n^{H840A} expression through the removal of thiamine; arresting cells in G2-M phase through reversible inhibition of Cdc2-*asM17* using the ATP analogue 3-BrB-PP1; releasing cells from the G2-M arrest by removal of 3-BrB-PP; and sampling cells for subsequent Southern blot analysis of genomic DNA. Cas9n^{H840A} expression is controlled by the thiamine repressible *nmt81* promoter which, under our experimental conditions, takes ~28 hours to fully de-repress following removal of thiamine.

(C and D) Detection of SSBs and DSBs in genomic DNA by Southern blot analysis. DNA was extracted from Cas9n^{H840A}/gRNA1 expressing cells sampled at the indicated times following release from a G2-M arrest. The DNA was cut with PstI and NheI and run on both a denaturing gel (C) and a native gel (D). The DNA was then transferred to a positively charged nylon membrane by Southern blotting and the relevant restriction fragment was detected using probe S. Southern blots from two independent experiments are shown side by side.
(E) Percentage of septating cells (septation index) following release from G2-M arrest. These data indicate a highly synchronous mitotic progression of cells following release from G2-M arrest. Note that *S. pombe* cells enter S-phase before completing septation. Data are presented as mean values ± SEM.
(F) Quantification of the Southern blots shown in (C) and (D). Data are presented as mean values ± SEM.

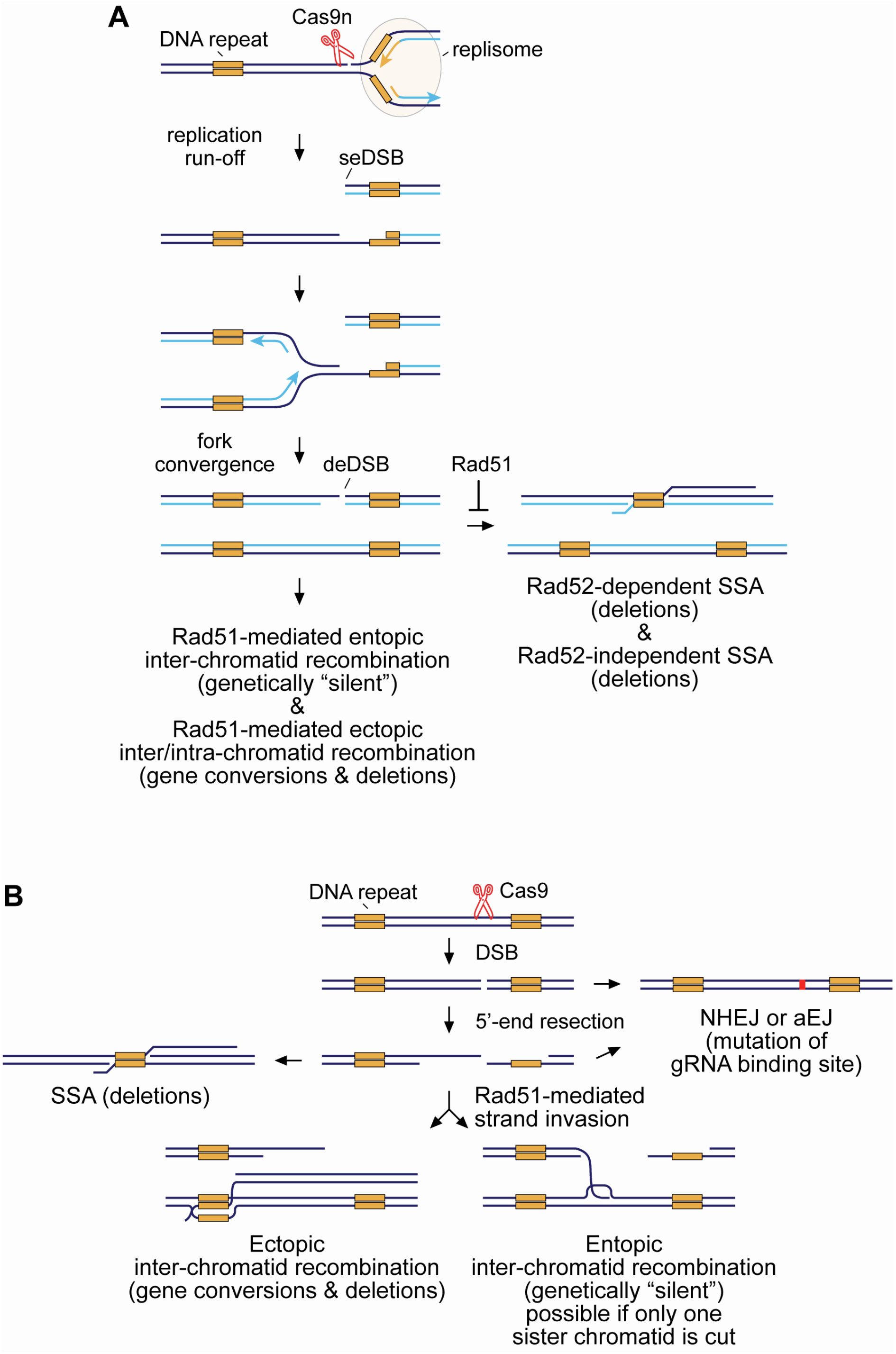

Figure S2. Models for the repair of DSBs induced by Cas9 and Cas9n, related to Figure 2.
(A) The repair of DSBs caused by replication fork encounters with Cas9n-induced SSBs. See main text for further details.
(B) The repair of Cas9-induced DSBs. Rad51-mediated ectopic intra-chromatid recombination is not shown but can also give rise to gene conversions and deletions. If both sister chromatids are cut by Cas9 then effective DSB repair by inter/intra-chromatid recombination will only generate deletions. See main text for further details.

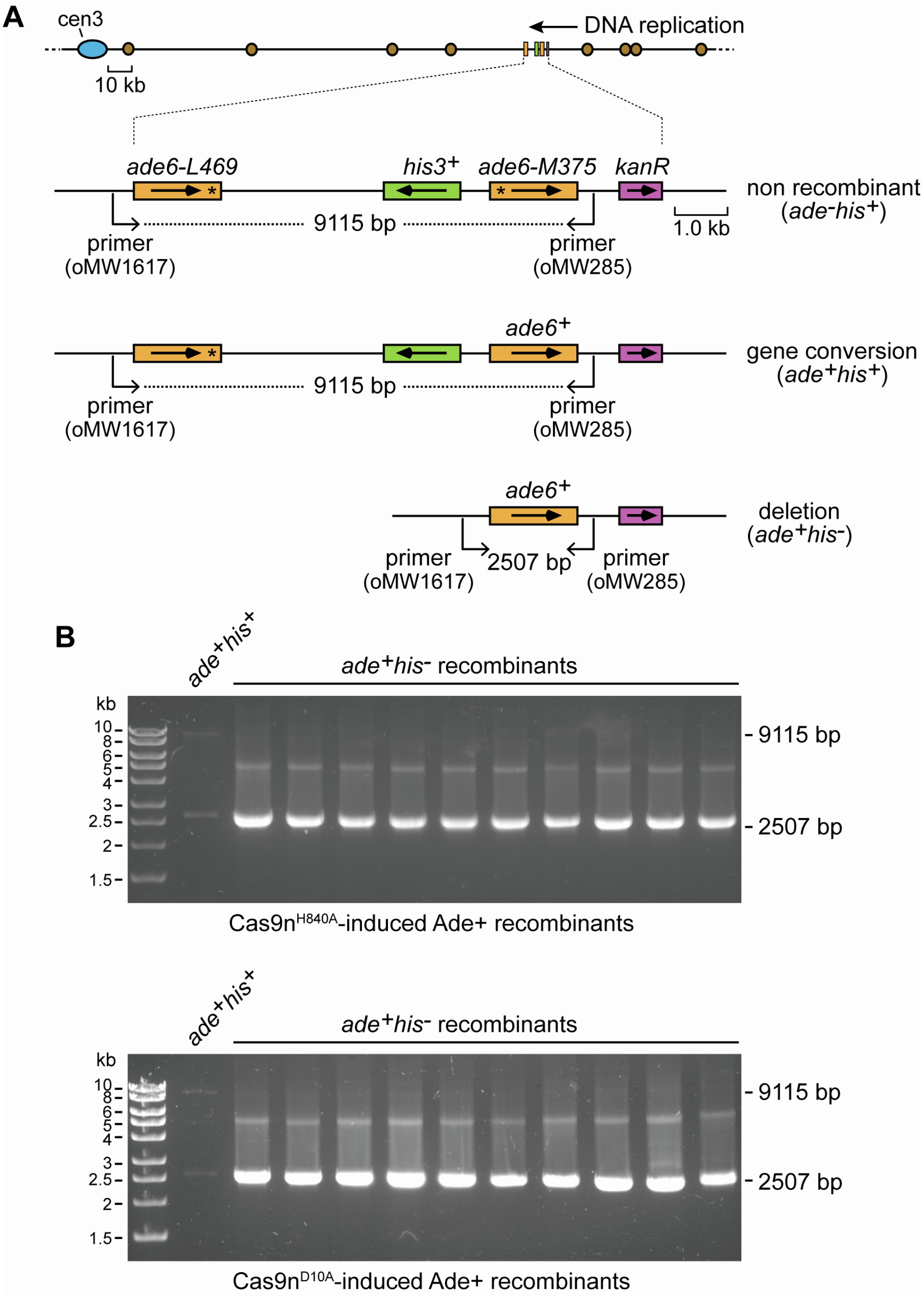

Figure S3. Colony PCR analysis of Cas9n-induced Ade+ recombinants, related to Figure 4.
(A) Diagram showing the configuration Ade+ recombinants that result from BIR-associated template switching at the *ade6*⁻ direct repeat reporter shown in Figure 3A. The position of primer binding sites and expected sizes of PCR products are indicated.
(B) Analysis of PCR products by agarose gel electrophoresis. Randomly selected Cas9n-induced Ade+ His+ and Ade+ His- recombinant colonies were subject to PCR analysis using primers oMW1617 and oMW285.

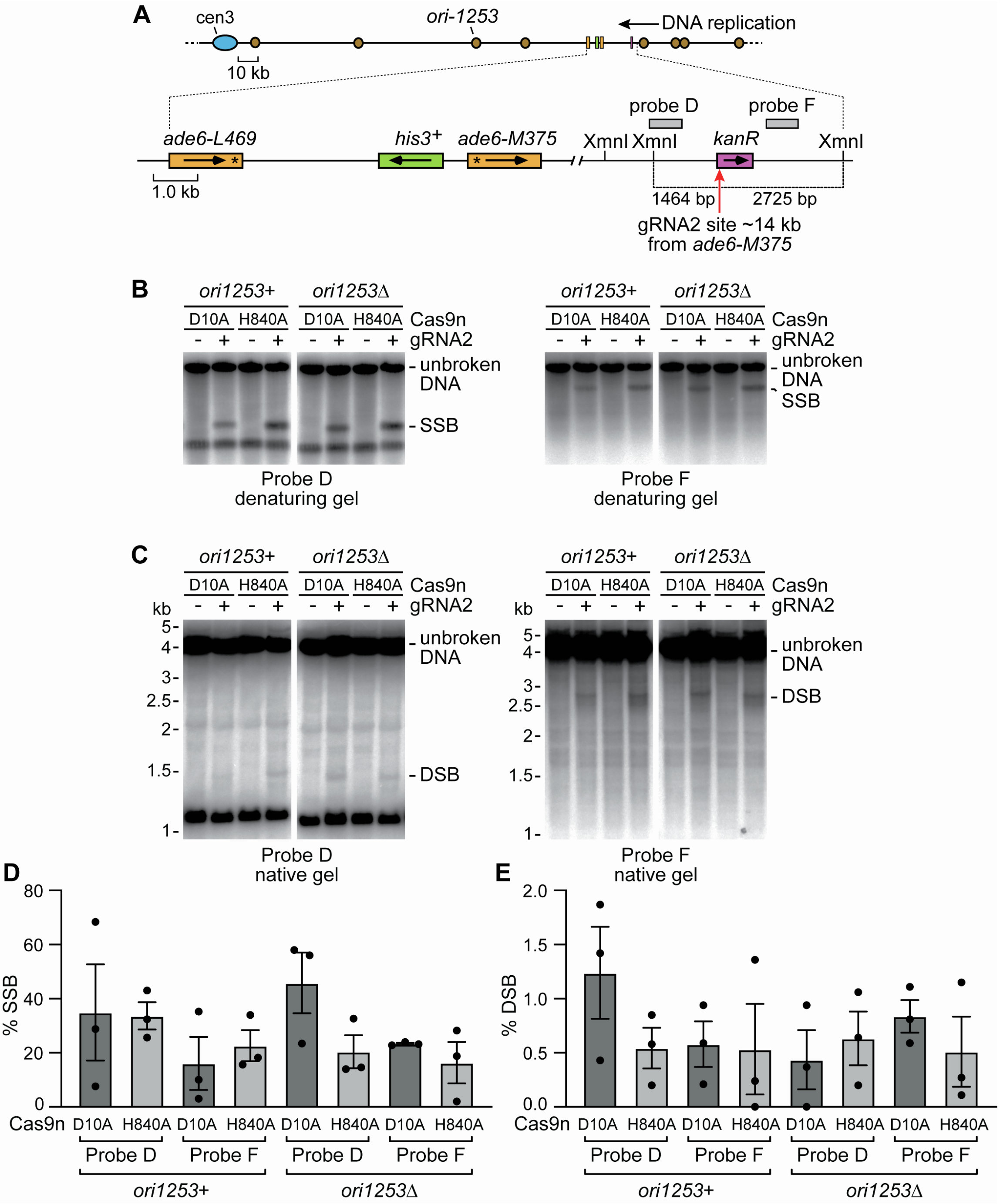

Figure S4. Detection of Cas9n-induced SSBs and deDSBs in *kan^R*, related to Figure 5.
(A) Diagram of the same recombination reporter shown in Figure 5A. Relevant restriction sites, probes (grey rectangles), and expected SSB/DSB DNA fragment sizes are indicated. The red arrow indicates the location of the gRNA2 binding site in *kan^R* (purple rectangle).
(B) Detection of SSBs in genomic DNA by Southern blot analysis. DNA was extracted from exponentially growing cultures of cells expressing the indicated version of Cas9n with and without gRNA2. The DNA was cut with XmnI, run on a denaturing gel, Southern blotted, and the relevant restriction fragment detected using probes D and F. This analysis was performed with strains with *ori1253* present and deleted.
(C) Detection of DSBs in genomic DNA by Southern blot analysis. The same XmnI digested DNA samples analysed in (B) were also run on native gels to detect DSBs.
(D) Quantification of the SSB signals detected by Southern blot analysis. Data are presented as mean values ± SD. Individual data points are shown as black dots.

(E) Quantification of the DSB signals detected by Southern blot analysis. Data are presented as mean values ± SD. Individual data points are shown as black dots.

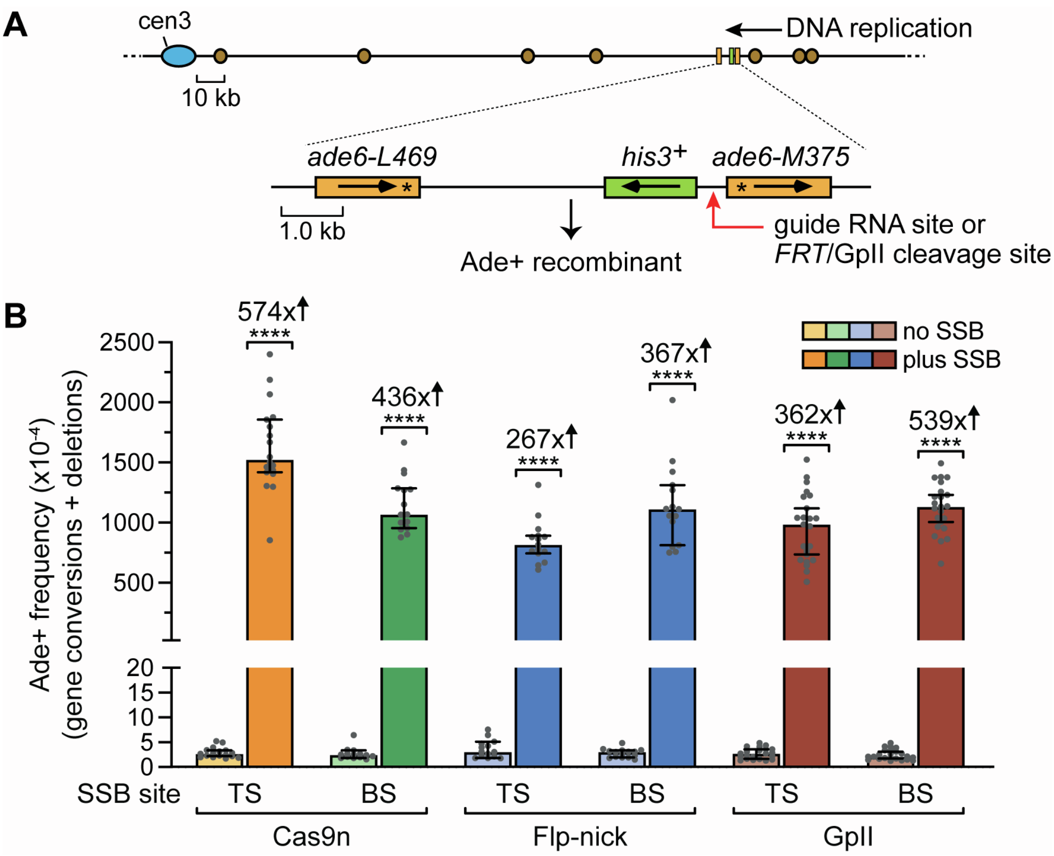

Figure S5. Cas9n, Flp-nick and gpII induce similar levels of direct repeat recombination, related to Figure 6.

(A) Diagram showing the location of the recombination reporter and SSB site (red arrow) on chromosome 3. Centromere (blue oval), replication origins (brown circles), and genes (orange and green rectangles with arrows indicating the direction of transcription) are depicted. Asterisks indicate the position of loss of function mutations in the two *ade6* alleles. Five versions of the recombination reporter were used that differed only at the SSB site, which varied to accommodate either a gRNA1 binding site, *FRT* site (both orientations) or gpII cleavage site (both orientations). Note that the location of the recombination reporter is the same as in Figure 1A. The only difference between this reporter and the one in Figure 1A is a shorter interval (by ~2 kb) between *his3* and *ade6-M375*.

(B) Frequency of Ade+ recombinants induced by Cas9n, Flp-nick and gpII in strains containing the recombination reporter shown in (A). The position of the cleavage site in either the leading template (top) strand (TS) or lagging template (bottom) strand (BS) is indicated. For Cas9n, its expression without gRNA1 acts as a no SSB control. Absence of the nicking enzyme acts as the no SSB control for Flp-nick and gpII. The data are presented as median values ± 95% confidence interval with individual data points shown as grey dots. The indicated fold changes and p values refer to the comparison of recombination values with equivalent no SSB control strains. The p values were calculated using the two-tailed Mann-Whitney test. **** p <0.0001. The data are also reported in Table S1, which includes the strain numbers, the number of colonies tested for each strain (n) and relevant p values.

Table S2: Mutation frequencies, Related to Figure 3

Relevant Genotype<br>plus strain no. and plasmid no.	Cas9 variant<br>and<br>gRNA2	Total number of colonies analysed in 5 independent experiments	% G418 sensitive colonies<br>(Mean ± SD)
wild-type<br>MCW10229 + pLX3	Cas9<br>no gRNA	2938	0 (± 0)
wild-type<br>MCW10229 + pLX32	Cas9<br>plus gRNA2	2990	75.6 (± 38.9)
wild-type<br>MCW10236 + pLX3	Cas9n^{H840A}<br>no gRNA	3080	0 (± 0)
wild-type<br>MCW10236 + pLX32	Cas9n^{H840A}<br>plus gRNA2	1345	0 (± 0)
wild-type<br>MCW10232 + pLX3	Cas9n^{D10A}<br>no gRNA	3096	0 (± 0)
wild-type<br>MCW10232 + pLX32	Cas9n^{D10A}<br>plus gRNA2	3078	0 (± 0)
rad51Δ<br>MCW11256 + pLX3	Cas9<br>no gRNA	1667	0 (± 0)
rad51Δ<br>MCW11256 + pLX32	Cas9<br>plus gRNA2	3895	99.8 (± 0.4)
rad51Δ<br>MCW10688 + pLX3	Cas9n^{H840A}<br>no gRNA	1792	0 (± 0)
rad51Δ<br>MCW10688 + pLX32	Cas9n^{H840A}<br>plus gRNA2	1938	0.53 (± 0.78)
rad51Δ<br>MCW10686 + pLX3	Cas9n^{D10A}<br>no gRNA	1497	0 (± 0)
rad51Δ<br>MCW10686 + pLX32	Cas9n^{D10A}<br>plus gRNA2	2025	0.26 (± 0.36)
rad51Δ rad52Δ<br>MCW11260 + pLX3	Cas9<br>no gRNA	1910	0 (± 0)
rad51Δ rad52Δ<br>MCW11260 + pLX32	Cas9<br>plus gRNA2	1185	99.8 (± 0.3)
rad51Δ rad52Δ<br>MCW10696 + pLX3	Cas9n^{H840A}<br>no gRNA	2512	0 (± 0)
rad51Δ rad52Δ<br>MCW10696 + pLX32	Cas9n^{H840A}<br>plus gRNA2	1581	0 (± 0)
rad51Δ rad52Δ<br>MCW10694 + pLX3	Cas9n^{D10A}<br>no gRNA	2868	0 (± 0)
rad51Δ rad52Δ<br>MCW10694 + pLX32	Cas9n^{D10A}<br>plus gRNA2	1281	0 (± 0)
ku70Δ<br>MCW11158 + pLX3	Cas9<br>no gRNA	1687	0.07 (± 0.16)
ku70Δ<br>MCW11158 + pLX32	Cas9<br>plus gRNA2	2964	100 (± 0)
ku70Δ<br>MCW10680 + pLX3	Cas9n^{H840A}<br>no gRNA	1881	0 (± 0)
ku70Δ<br>MCW10680 + pLX32	Cas9n^{H840A}<br>plus gRNA2	2238	0 (± 0)
ku70Δ<br>MCW10678 + pLX3	Cas9n^{D10A}<br>no gRNA	2329	0 (± 0)
ku70Δ<br>MCW10678 + pLX32	Cas9n^{D10A}<br>plus gRNA2	1369	0 (± 0)
rad51Δ rad52Δ ku80Δ<br>MCW11296 + pLX3	Cas9<br>no gRNA	1962	0 (± 0)
rad51Δ rad52Δ ku80Δ<br>MCW11296 + pLX32	Cas9<br>plus gRNA2	1036	80 (± 44.7)
rad51Δ rad52Δ ku80Δ	Cas9n^{H840A}	1097	0 (± 0)

MCW11266 + pLX3	no gRNA		
rad51Δ rad52Δ ku80Δ MCW11266 + pLX32	Cas9n^{H840A} plus gRNA2	1962	0 (± 0)
rad51Δ rad52Δ ku80Δ MCW11270 + pLX3	Cas9n^{D10A} no gRNA	1361	0 (± 0)
rad51Δ rad52Δ ku80Δ MCW11270 + pLX32	Cas9n^{D10A} plus gRNA2	1987	0 (± 0)

Table S4: Oligonucleotides, Related to STAR Methods

Oligo No.	Sequence (5' – 3')
oMW1708	AAGCTTATCGATACCGTCGAGT
oMW1709	TCGACGGTATCGATAAGCTTTT
oMW2158	GTCTTTTCCTTTCTTCGGTACAGGTTATG
oMW2159	TCACGTTTCGGTTTTAGAGCTAGAAATAGC
oMW480	TTTAAAGTCGACCTCGAGGTTCTTTAATAGTGGACTCTTGTTCCAAACTGGA-ACAACACCCGGGTTTAAA
oMW481	TTTAAACCCGGGTGTTGTTCCAGTTTGGAACAAGAGTCCACTATTAAAG-AACCTCGAGGTCGACTTTAAA
oMW482	TTTAAACCCGGGCTCGAGGTTCTTTAATAGTGGACTCTTGTTCCAAACTG-GAACAACAGTCGACTTTAAA
oMW483	TTTAAAGTCGACTGTTGTTCCAGTTTGGAACAAGAGTCCACTATTAAAGA-ACCTCGAGCCCGGGTTTAAA
oMW2031	ATCGAAGTTCCTATACTTTCTAGAGAATAGGAACTTCCGAATAGGAACTTCG
oMW2032	TCGACGAAGTTCCTATTCGGAAGTTCCTATTCTCTAGAAAGTATAGGAACTTCGAT
oMW2068	ATCGAAGTTCCTATTCGGAAGTTCCTATTCTCTAGAAAGTATAGGAACTTCG
oMW2069	TCGACGAAGTTCCTATACTTTCTAGAGAATAGGAACTTCCGAATAGGAACTTCGAT
oMW2029	GGGTCGACATGCCACAATTTGGTATATTATGTA
oMW2030	TAGGATCCTAATTATATGCGTCTATTTATGTAGGATGAAA
oMW285	AATGTACGGGCGACAGTC
oMW1617	TATCAGCTGTACTTATTTACGTACTGTG
oMW706	AAAGGCCTCGCTTCTCGAG
oMW707	AGCAGCATACGCTAAAATC
oMW705	AAGAACCTACTGAGCCTACG
oMW1627	TTAGAGCTCCGAATAATGTGCTGCGACG

References

[S1] Jalan, M., Oehler, J., Morrow, C.A., Osman, F., and Whitby, M.C. (2019). Factors affecting template switch recombination associated with restarted DNA replication. Elife *8*, e41697. https://doi.org/10.7554/eLife.41697.

[S2] Osman, F., Ahn, J.S., Lorenz, A., and Whitby, M.C. (2016). The RecQ DNA helicase Rqh1 constrains Exonuclease 1-dependent recombination at stalled replication forks. Sci. Rep. *6*, 22837. https://doi.org/10.1038/srep22837.
